# Morphological Structure Identification, Comparative Mitochondrial Genomics and Population Genetic Analysis toward Exploring Interspecific Variations and Phylogenetic Implications of *Malus baccata* ‘ZA’ and Other Species

**DOI:** 10.3390/biom14080912

**Published:** 2024-07-26

**Authors:** Xun Wang, Daru Wang, Ruifen Zhang, Xin Qin, Xiang Shen, Chunxiang You

**Affiliations:** 1 Apple Technology Innovation Center of Shandong Province, Shandong Collaborative Innovation Center of Fruit & Vegetable Quality and Efficient Production, National Key Laboratory of Wheat Improvement, College of Horticultural Science and Engineering, Shandong Agricultural University, Taian 271018, China; 2021010063@sdau.edu.cn (X.W.); 2020110302@sdau.edu.cn (D.W.); 2021110277@sdau.edu.cn (X.Q.); 2 Qingdao Apple Rootstock Research and Development Center, Qingdao Academy of Agricultural Sciences, Qingdao 266100, China; zhangruifen316@gmail.com

**Keywords:** Rosaceae, dwarf mutant, collinearity analysis, repeat sequences, mitochondrial plastid DNAs, codon usage bias

## Abstract

*Malus baccata*, a valuable germplasm resource in the genus *Malus*, is indigenous to China and widely distributed. However, little is known about the lineage composition and genetic basis of ‘ZA’, a mutant type of *M. baccata*. In this study, we compared the differences between ‘ZA’ and wild type from the perspective of morphology and ultrastructure and analyzed their chloroplast pigment content based on biochemical methods. Further, the complete mitogenome of *M. baccata* ‘ZA’ was assembled and obtained by next-generation sequencing. Subsequently, its molecular characteristics were analyzed using Geneious, MISA-web, and CodonW toolkits. Furthermore, by examining 106 *Malus* germplasms and 42 Rosaceae species, we deduced and elucidated the evolutionary position of *M. baccata* ‘ZA’, as well as interspecific variations among different individuals. In comparison, the total length of the ‘ZA’ mitogenome (GC content: 45.4%) is 374,023 bp, which is approximately 2.33 times larger than the size (160,202 bp) of the plastome (GC: 36.5%). The collinear analysis results revealed abundant repeats and genome rearrangements occurring between different *Malus* species. Additionally, we identified 14 plastid-driven fragment transfer events. A total of 54 genes have been annotated in the ‘ZA’ mitogenome, including 35 protein-coding genes, 16 tRNAs, and three rRNAs. By calculating nucleotide polymorphisms and selection pressure for 24 shared core mitochondrial CDSs from 42 Rosaceae species (including ‘ZA’), we observed that the *nad3* gene exhibited minimal variation, while *nad4L* appeared to be evolving rapidly. Population genetics analysis detected a total of 1578 high-quality variants (1424 SNPs, 60 insertions, and 94 deletions; variation rate: 1/237) among samples from 106 *Malus* individuals. Furthermore, by constructing phylogenetic trees based on both *Malus* and Rosaceae taxa datasets, it was preliminarily demonstrated that ‘ZA’ is closely related to *M. baccata*, *M. sieversii*, and other proximate species in terms of evolution. The sequencing data obtained in this study, along with our findings, contribute to expanding the mitogenomic resources available for Rosaceae research. They also hold reference significance for molecular identification studies as well as conservation and breeding efforts focused on excellent germplasms.

## 1. Introduction

*Malus baccata* (L.) Borkh., commonly known as ‘shanjingzi’, is a deciduous fruit tree indigenous to China. It belongs to the *Malus* genus (Rosaceae, Maloideae) and is extensively distributed throughout various regions of China, including Northeast China (Heilongjiang, Jilin, and Liaoning province), North China (Nei Mongol, Hebei, and Shanxi), and Northwest China (Shaanxi and Gansu province). This wide distribution can be attributed to its preference for sunlight, tolerance to cold temperatures, and adaptability characteristics [1,2,3]. Apart from China, this species can also be found in countries such as Russia and North Korea in North and East Asia [4]. The branches and leaves of *M. baccata* are lush, with a flowering period typically occurring from April to June. The fruits mature between September and October. Its tree posture, leaf shape, and flower coloration, as well as its fruit coloration, contribute to its exceptional ornamental value. Furthermore, it holds significant economic importance within the apple industry, where it is utilized for rootstock or variety enhancement purposes [5].

Due to the wide distribution of *M. baccata* and its diverse adaptability to different living environments and ecological conditions, a multitude of varieties and variants have been discovered in various regions of China [6,7], thereby further enriching the germplasm diversity of *M. baccata* and *Malus*. For instance, common variations such as *M. baccata* f. *gracilis* Rehd., var. *latifolia* Skv., and f. *villosa* Skv. have been reported [8,9]. In 1976, Chinese scientists identified a dwarf mutation type called *M. baccata* ‘ZA’ from ‘shanjingzi’ in Hulunbuir City, Nei Mongol Autonomous Region [10,11,12]. This germplasm exhibits exceptional cold resistance, with stable dwarfish genetic traits controlled by a dominant major gene. Consequently, this valuable mutation resource of *M. baccata* holds significant advantages for cross-breeding *Malus* and apple cultivars with dwarfism and enhanced resistance [10]. However, its evolutionary origins within the genus *Malus* and its biological role within the family Rosaceae remain poorly understood, impeding research progress on *M. baccata* ‘ZA’.

In the study of molecular phylogeny and population inheritance, the mitogenome possesses unique advantages [13,14,15]. As a crucial component of maternal inheritance, the mitogenome exhibits a relatively short length and gene conservation, rendering it an exceptional molecular dataset [16]. However, due to its intricate structure and abundance of exogenous sequences and repetitive fragments, obtaining the complete sequence is challenging [17]. With advancements in sequencing technology and assembly tools, numerous plant mitogenomes have been released in recent years [17,18,19], providing vital support for species traceability and genetic breeding. Currently, there are over 100 Rosaceae mitogenomes available in the NCBI database, with approximately ten belonging to *Malus* species (including *M. domestica* (Suckow) Borkh., *M. sieversii* (Ledeb.) M. Roem., *M. sylvestris* (L.) Mill., *M. hupehensis* (Pamp.) Rehder, *M. baccata*). It should be noted that apart from the aforementioned *M. baccata*, the *Malus* genus encompasses more than thirty other species as well [8,20,21,22,23], such as *M. asiatica* Nakai, *M. prunifolia* (Willd.) Borkh., *M. micromalus* Makino, *M. sieboldii* Rehder, and *M. yunnanensis* (Franch.) C. K. Schneid. It is impossible to elucidate the complex interspecific relationships of *Malus* with the limited genomic data. Decoding the mitogenome of the valuable *Malus* germplasm ‘ZA’ can not only unravel its identity mystery and increase available resources for the database but can also hold far-reaching importance for elucidating the evolution of *Malus* and Rosaceae.

The complete mitogenome of *M. baccata* ‘ZA’ was assembled and annotated based on next-generation sequencing and reference datasets in this study. Furthermore, the analysis was conducted on its genome composition, intraspecific and interspecific collinearity, distribution of repeat sequences, and sequence migration events. Additionally, the population evolution of *Malus* and the molecular phylogeny of Rosaceae were discussed by integrating resequencing data with other mitogenome maps. Consequently, a detailed comparison of these datasets establishes a reliable foundation for the conservation and utilization of the ‘ZA’ dwarf mutant.

## 2. Materials and Methods

### 2.1. Material Collection, Sample Extraction, and DNA Sequencing

*Malus baccata* ‘ZA’ for morphological identification and mitogenome assembly was cultivated at Shandong Agricultural University, National Apple Engineering Technology Research Center (36.162410° N, 117.157452° E, Taian, Shandong, China), and subjected to standard agronomic measures for daily management during growth. For ultrastructure detection, scanning electron microscopy (Regulus 8100, Hitachi, Tokyo, Japan) was used for imaging, in which the plant leaves were fixed with a glutaraldehyde solution. The content of photosynthetic pigments (chlorophylls and carotenoids) was determined by spectrophotometric method (95% ethanol was used as blank, absorbance was recorded at the wavelength of 665 nm, 649 nm, and 470 nm), and chlorophyll was extracted and separated by organic solvent ethanol. In addition, young leaves free from pests were collected in the morning on a clear day and immediately frozen in a liquid nitrogen storage tank. They were then temporarily stored in an ultra-low-temperature refrigerator at −80 °C for subsequent experimental arrangements. The cetyl trimethyl ammonium bromide (CTAB) method was employed to extract tissue DNA from the samples, and DNA quality was detected by agarose gel electrophoresis. The whole-genome sequencing of *M. baccata* ‘ZA’ was completed using the Hiseq-Xten PE150 platform (Illumina Inc., San Diego, CA, USA) and was supported by the Novogene Bioinformatic Technology Co., Ltd. (Tianjin, China). For Illumina sequencing, the paired-end library (2 × 150 bp) was constructed with an insert size of 350 bp. Additionally, 105 germplasm materials of *Malus* spp., including *M. domestica*, *M. sieversii*, *M. sylvestris*, *M. hupehensis*, *M. baccata*, *M. sieboldii*, *M. yunnanensis*, *M. toringoides* (Rehder) Hughes, *M. tschonoskii* (Maxim.) C. K. Schneid., and *M. ioensis* (Alph. Wood) Britton, were used for population evolution analysis in this study (Table S1). These species were planted at Qingdao Academy of Agricultural Sciences, Qingdao Apple Rootstock Research and Development Center (36.238269° N, 120.539478° E, Qingdao, Shandong, China). The sampling methods, as well as extraction and sequencing procedures, remained consistent with ‘ZA’.

### 2.2. Sequencing Data Processing and Mitochondrial Genome Assembly

The Illumina reads in raw data were filtered based on the following criteria: (1) removal of reads containing sequencing adapters; (2) removal of reads with an unknown base ratio greater than 10%; (3) removal of reads containing more than 20% low-quality bases. For the *M. baccata* ‘ZA’ mitogenome, the assembly strategy adopted was as follows: De novo assembly of the mitogenome was accomplished using Unicycler software (v0.5.0) [24]. In Unicycler, SPAdes initial assembly was performed based on K-mer values (27, 53, 71, 87, 99, 111, 119, and 127), followed by SPAdes contigs creation, loop unrolling bridges formation, and bridges application for assembly graph construction and structure simplification (Figure S1) [25]. The assembly results were visualized using Bandage version-0.8.1 [26]. Additionally, the integrity of the mitogenome (Figure S2) was confirmed through read coverage analysis (BWA 0.7.17, SAMtools 1.16, and BAMStats 0.3.5) [27,28]. To further assess the quality of ‘ZA’ mitogenome assembly in this study, the core protein-coding genes (PCGs) were annotated below, which also serves as evidence for result reliability (no possible missing PCGs found). Finally, the complete mitogenome sequence of *M. baccata* ‘ZA’ was submitted to the NCBI database, and the entry number (PP826182) was obtained. The raw data used for assembly are stored in the Genome Sequence Archive (GSA) at the National Genomics Data Center (CRA016093, https://ngdc.cncb.ac.cn/, accessed on 15 March 2024) [29,30]. Furthermore, using canu [31], we assembled the mitogenome (OR876282) of an apple cultivar (*M. domestica* ‘Honeycrisp’) from the SRA sequencing dataset available in the NCBI database [32,33]. For subsequent comparative analysis of mitogenomes, both newly obtained and previously published sequences were utilized (Table S2).

### 2.3. Annotation of Coding Sequence, Transfer RNA, and Ribosomal RNA in Mitogenome

Gene identification of the ‘ZA’ mitogenome was performed using the GeSeq (https://chlorobox.mpimp-golm.mpg.de/geseq.html, accessed on 30 March 2024) and IPMGA tool (http://www.1kmpg.cn/ipmga/, accessed on 1 April 2024) [34,35,36]. Three *Malus* mitogenomes (RefSeq: NC_065224, NC_065225, NC_065226) and the angiosperm mitogenome + 43-plastome dataset were selected as references for annotation. The annotation genes include coding sequences (CDSs), transfer RNA (tRNA), and ribosomal RNA (rRNA). For tRNA identification, the results from ARAGORN v1 tool and tRNAscan-SE 2.0 tool were integrated. Manual confirmation was conducted to verify the annotated information of all genes, particularly those with multiple introns. The PMGmap toolkit (http://47.96.249.172:16086/drawing/, accessed on 1 April 2024) was utilized for drawing and visualization of the mitogenome map of *M. baccata* ‘ZA’ [37].

### 2.4. Mitogenome Composition, Codon Usage Bias, and Collinearity Analysis

The mitogenome comprises tandem repeats (short tandem repeats—STRs, long tandem repeats—LTRs) and scattered repeats (dispersed repeats—DRs). Among them, STRs were obtained using MISA-web (https://webblast.ipk-gatersleben.de/misa/, accessed on 5 April 2024), LTRs were calculated on the TRF website, and REPuter (https://bibiserv.cebitec.uni-bielefeld.de/reputer, accessed on 5 April 2024) was utilized for analyzing dispersed repeats with a minimal repeat size of 30 and a hamming distance of 3. It should be noted that the motif repetitions identified by MISA-web were specified as follows: 10 repetitions for 1 bp, 5 repetitions for 2 bp, 4 repetitions for 3 bp, and 3 repetitions each for 4 bp, 5 bp, and 6 bp motifs; all other options were selected in default mode. Additionally, the characteristics of mitogenomes also include GC statistics, such as GC content ([nG + nC]/[nA + nT + nG + nC]) and GC skew ([nG − nC]/[nG + nC]), which are determined using CGView 1.0.2 software (https://stothardresearch.ca/cgview/, accessed on 5 April 2024) [38,39]. The sliding window algorithm is employed for these calculations with a window size of 1000 and a step value of 10. According to the annotations, the coding sequences were extracted from the mitogenomes using Geneious R9 software [40]. Subsequently, their codon usage was characterized by employing the CodonW program, and the analysis indexes primarily encompassed codon adaptation index (CAI), codon bias index (CBI), effective number of codons (ENC), frequency of optimal codons (FOP), and relative synonymous codon usage (RSCU). The similarity analysis of mitogenomes in *M. baccata* ‘ZA’ and *M. baccata* was conducted using Geneious R9. Mitogenome collinearity and rearrangement of *Malus* species were performed using the Geneious process, employing the genome-wide comparison model (Mauve, progressive algorithm) [41,42].

### 2.5. Identification of Mitochondrial Plastid DNAs and Exchange of Organelle Fragments

About 5 Gb reads were randomly extracted from the sequencing results (SeqKit version-2.3.0), and then the plastid genome of *M. baccata* ‘ZA’ was assembled using Unicycler v0.5.0 and GetOrganelle v1.7.7.0 (parameters: -R 15; -k 21, 45, 65, 85, 105, 121, 127) [43]. The complete sequence of the resolved plastome was submitted to the CPGAVAS2 toolkit (http://47.96.249.172:16019/analyzer/home, accessed on 25 March 2024) for basic annotation, including gene types and repeat sequences [44]. The reference genomes NC_045389.1, KX499859.1, MK571561.1, OM232791.1, and OM232793.1 (*M. baccata* plastomes) were selected for analysis in this toolkit. The combined results were further examined to confirm the completeness and accuracy of gene structure using the Chloroplast Genome Viewer (CPGView, http://47.96.249.172:16085/cpgview/home, accessed on 25 March 2024) [45]. The assembled plastid genome sequence in FASTA format and its annotation in GenBank format were submitted to the NCBI website with accession number OR876281 for public access. The CPGView website was utilized to visualize the circular chloroplast genome. The identification of transfer fragments in both the plastome and mitogenome was based on homologous sequences with an E-value < 1 × 10^−6^ using BLASTN 2.12.0+; these fragments were considered mitochondrial plastid DNAs (MTPTs) [46]. The characteristic information of transferred fragments was extracted from annotated genomes. BLAST results were visualized using the R package circlize (https://jokergoo.github.io/circlize/, accessed on 25 March 2024) [47].

### 2.6. Population Evolution Based on Mitochondrial Genome

The collection of 105 *Malus* germplasm samples was conducted artificially, followed by whole-genome resequencing. For population genetic analysis, the mitogenome of *M. baccata* ‘ZA’ was used as a reference. BWA 0.7.15, samtools 1.6, and GATK v4.3 were employed for genome index construction, while quality control of raw data was performed using Trimmomatic program (version 0.39). Variation detection was carried out using GATK version 4.3 [48], with successive execution of HaplotypeCaller, CombineGVCFs, and GenotypeGVCFs modules. VCFtools (v0.1.16) was utilized to filter the original variations (-max-missing 0.8, -maf 0.05), resulting in high-quality variants [49]. Mutation site annotation, including single-nucleotide polymorphisms and insertions/deletions, was analyzed using SnpEff version 4.3 [50]. The construction of a population evolutionary tree based on variants (SNPs/INDELs) involves the following programs: BCFtools (v1.15.1), VCF2Dis version 1.47, FastME 2.1.6.4 (utilized for distance algorithms to infer phylogenies, https://gite.lirmm.fr/atgc/FastME/, accessed on 5 April 2024), and MEGA (Version X).

### 2.7. Phylogenetic Relationship and Interspecific Variation of Rosaceae

In order to elucidate more detailed species clustering and phylogenetic relationships, in addition to the mitogenome sequences of *Malus* (including *M. domestica*, *M. baccata*, *M. sieversii*, and *M. sylvestris* obtained from NCBI RefSeq: NC_018554.1, NC_065224.1, NC_065225.1, and NC_065226.1, respectively; as well as the reference sequence for *M. baccata* ‘ZA’ provided in this study—PP826182), we queried and downloaded the mitogenomes of other genera and species within Rosaceae from the NCBI RefSeq database (Table S2). Firstly, 24 shared protein-coding genes were extracted from these mitogenomes using Geneious R9. Then, their nucleotide diversity (Hd, Pi) and selection pressure (nonsynonymous_Ka and synonymous_Ks substitution rates) were calculated using DnaSP software (v6) [51]; subsequently, this allowed for a preliminary comparison of interspecific variations among 42 Rosaceae species, including *M. baccata* ‘ZA’. Data statistics and visualization were performed using WPS Office 2024 and ChiPlot v1. Then, through sequence alignment in Codon mode, followed by pruning and concatenation steps conducted with PhyloSuite v1.2.2, MAFFT v7 and Gblocks 0.91b [52], a dataset suitable for evolutionary analysis was generated. Finally, the reconstructed topology of the aforementioned sequence set was inferred using two types of phylogenetic methods: maximum likelihood (ML) and Bayesian inference (BI). The ML tree analysis was performed using IQ-TREE 2.2.6 with the following options: -m MFP for model selection, -b 1000 for bootstrap support estimation, and -alrt 1000 for SH-aLRT support estimation. The resulting tree was validated using both bootstrap and SH-aLRT support values. The outgroup of the unrooted tree was generated based on the first species in multiple sequence alignment (*Geum urbanum*). Bayesian inference was conducted using MrBayes 3.2.6 with the following settings: (lset nst = 6 rates = gamma mcmc ngen = 10,000,000 printfreq = 1000 samplefreq = 1000 nchains = 4 nruns = 2 burninfrac = 0.25 sumt contype = allcompat). Convergence of the MCMC process (Markov Chain Monte Carlo) was assessed based on the average standard deviation of split frequencies (ASDSF < 0.01), effective sample size (ESS > 200), and potential scale reduction factor (PSRF ≈ 1). Finally, Adobe Illustrator CS6 and FigTree version 1.4.4 were used to further refine and annotate the phylogenetic trees.

## 3. Results

### 3.1. Morphological and Physiological Characteristics of M. baccata ‘ZA’

**Figure 1:**
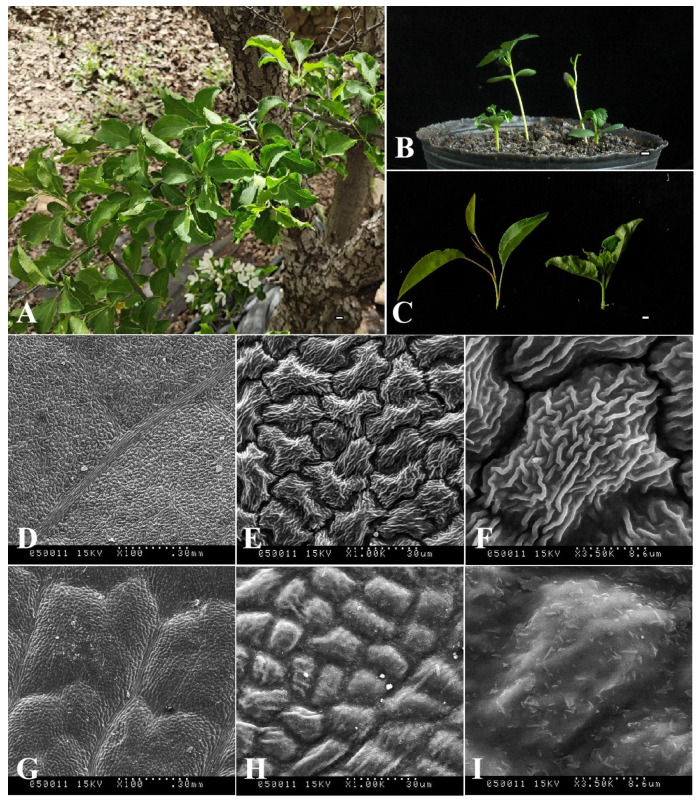
Morphological structure identification of *M. baccata* ‘ZA’ and wild type (*M. baccata*, MB). (**A**) Appearance of the ‘ZA’ mutant (stems, leaves, and flowers). (**B**) Growth status and plant height of ‘ZA’ and MB seedlings. The middle two plants are WT and the outer two are ‘ZA’. (**C**) Leaf morphological characteristics of two types—WT on the left and ‘ZA’ on the right. For (**A**–**C**), the white short line represents 1 cm. (**D**–**I**) Comparison of ultrastructure difference of upper epidermis between MB (**D**–**F**) and ZA (**G**–**I**). Each sample is displayed at three magnifications (100×, 1000×, and 3500×).

In order to more clearly define the morphological differences between the ‘ZA’ mutation type and wild type (*M. baccata*, MB), their plant heights and leaf tissues were compared (Figure 1). As shown in Figure 1B, the height of ‘ZA’ seedlings was significantly lower than that of wild type (WT), accounting for about one-third. Further observation showed that the leaves of ‘ZA’ were folded and curved (Figure 1A–C), which was significantly different from WT (Figure 1C). Based on scanning electron microscopy, the ultrastructures of these two kinds of leaves (‘ZA’ and MB) were analyzed (Figure 1D–I). The results showed that in three different visual fields, the cuticle of the ‘ZA’ leaf was significantly thickened (Figure 1G–I). It should be noted that ‘ZA’ has more epidermal wax than MB, a phenomenon that can be easily distinguished at 3500× magnification (Figure 1F,I).

**Figure 2:**
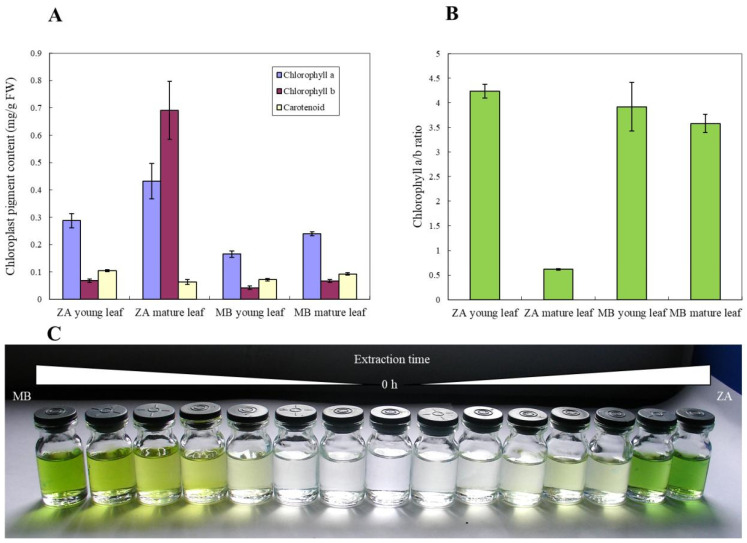
Photosynthetic pigment characteristics of *M. baccata* ‘ZA’ and wild type MB. (**A**) Chlorophyll a/b and carotenoid contents of young and mature leaves in ‘ZA’ and MB. (**B**) Chlorophyll a/b values of young and mature leaves in ‘ZA’ and MB. (**C**) The difference in chlorophyll extraction time between two plants (MB and ‘ZA’). The interval is one quarter hour.

Since the histomorphology of ‘ZA’ and WT leaves showed significant differences, chloroplast photosynthetic pigments were also used for comparison (Figure 2). As can be seen from Figure 2A, the photosynthetic pigment content in ‘ZA’ leaves is higher, and the chlorophyll content in mature leaves of ‘ZA’ and MB is higher than that in young leaves (Figure 2A). In the four experimental groups tested, the content of chlorophyll a and b in mature leaves of ‘ZA’ is much higher than that in the other three groups (Figure 2A). In addition, the content of chlorophyll b in the mature leaves of ‘ZA’ is higher than that of chlorophyll a, which is opposite to other comparisons, and this phenomenon can also be observed in the chlorophyll a/b ratio (Figure 2B). However, although the chlorophyll content of *M. baccata* ‘ZA’ is high, it is difficult and time-consuming to extract it (Figure 2C), reflecting its unique biological characteristics.

### 3.2. Basic Characteristics and Annotations of Malus baccata ‘ZA’ Mitogenome

To explore the lineage composition and genetic clues of *M. baccata* ‘ZA’ and other *Malus* plants, we assembled the complete mitogenome of ‘ZA’. The size is 374,023 bp (master circle structure), which is the smallest among the compared *Malus* species (Figure S1 and Table 1). By aligning the original reads to the mitogenome, the sequencing depth was calculated (mean 604.43×), which can be used for subsequent analysis (Figure S2 and Table S3). Additionally, we assembled the mitogenome of another cultivated apple variety, *M. domestica* ‘Honeycrisp’, which had a sequence length of 396,949 bp (Table 1). Despite variations in mitogenome size among different *Malus* species, including *M. baccata* ‘ZA’ ranging from 374,023 bp to 453,068 bp (*M.* ‘SH6’), their GC content remained relatively consistent within a range of 45.0%~45.5%, with most species having a GC content of approximately 45.4% (Table 1). Similarly, for the ‘ZA’ mitogenome, its GC% was also determined as 45.4% based on an analysis of base composition, where G/C bases accounted for a total length of 169,691 bp out of its entire sequence length (85,435 + 84,256 = 169,691 bp). In addition to GC content, G/C skew values (G/C base bias in single-stranded DNA) were calculated and compared across 10 *Malus* species mitogenomes (Figure S3 and Table 1), revealing that this value ranged from −0.2695 to 0.2706 in *M. baccata* ‘ZA’, with an average value being 0.006173, which showed a slight difference when comparing it with that in *M. baccata* (−0.2739~0.2682, 0.007541) (Table 1).

**Figure 3:**
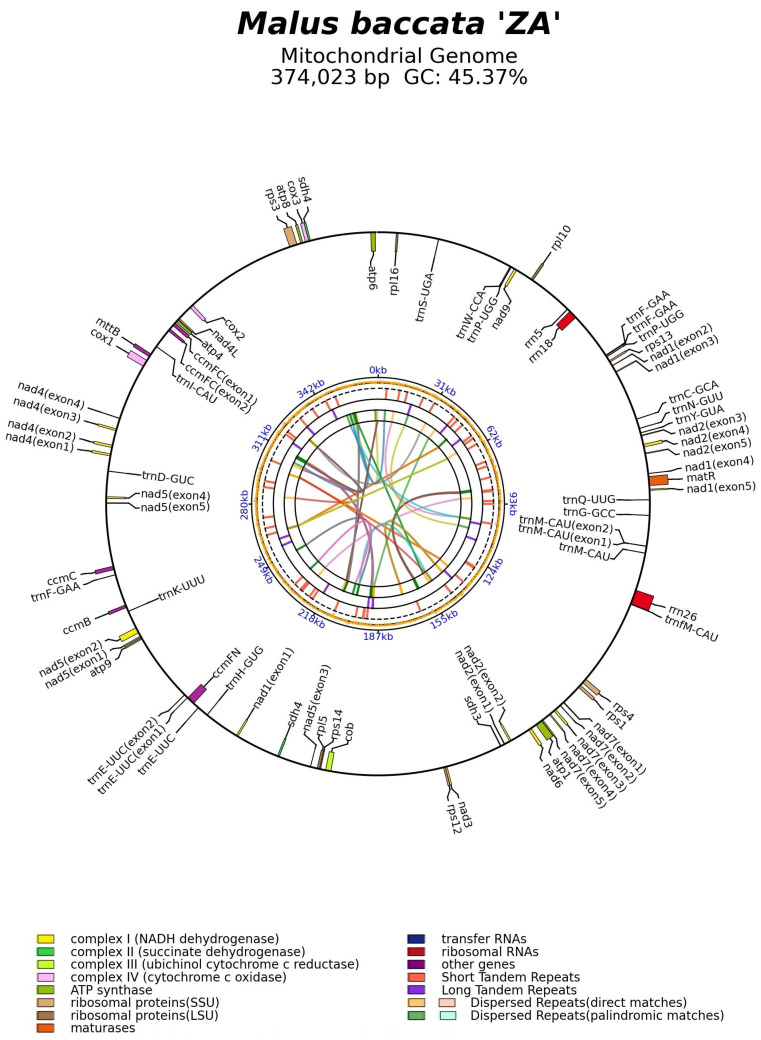
The mitogenome map of *M. baccata* ‘ZA’. The shaded parts (orange) in the figure represent the GC content of each region of the genome. Different classes of mitochondrial genes are represented by different colors, and annotated genes with introns are marked with parentheses. The short lines in the inner circle represent the repeat sequence of the mitogenome.

The mitogenome of *M. baccata* ‘ZA’ was annotated using the reference datasets, resulting in the identification of 54 genes (including 35 CDSs, 16 tRNAs, and 3 rRNAs) (Figure 3). As shown in Table 2, the set of 35 PCGs can be further categorized into two groups: core genes (*atp1*, *atp4*, *atp6*, *atp8*, *atp9*, *ccmB*, *ccmC*, *ccmFC*, *ccmFN*, *cob*, *cox1*, *cox2*, *cox3*, *matR*, *mttB*, *nad1*, *nad2*, *nad3*, *nad4*, *nad4L*, *nad5*, *nad6*, *nad7*, and *nad9*) and variable genes (*rpl5*, *rpl10*, *rpl16*, *rps1*, *rps3*, *rps4*, *rps12*, *rps13*, *sdh3*, and *sdh4*). The locations and specific details of these genes are listed in Table S4. Among all the identified genes, six CDSs (*ccmFC*, *nad1*, *nad2*, *nad4*, *nad5*, and *nad7*), as well as two tRNAs (*trnE-UUC* and *trnM-CAU*), contain introns (Figure 3, Figures S4 and S5, Tables S4 and S5). Notably, the *trans*-splicing phenomenon is observed in three particular genes (*nad1*, *nad2*, and *nad5*) (Figure 3 and Figure S5).

### 3.3. Repeat Sequences in Mitochondrial Genomes of M. baccata ‘ZA’ and Other Malus Species

**Figure 4:**
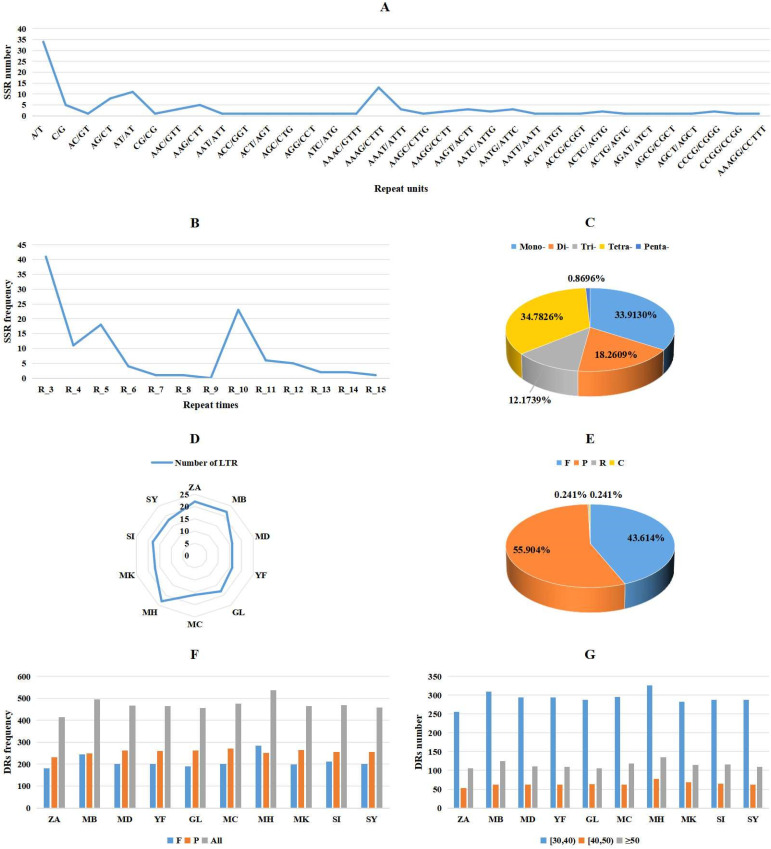
Identification and comparison of mitogenome repeats in *M. baccata* ‘ZA’ and nine *Malus* species. (**A**,**B**) Frequency statistics of SSRs with different repeat units and repeat times in ‘ZA’ mitogenome. (**C**) The proportion of the five SSRs (mono-, di-, tri-, tetra-, and penta-) in ‘ZA’ mitogenome. (**D**) Comparison of the number of LTRs in mitogenomes of 10 *Malus* species. (**E**) Four different classes of DR repeats in ‘ZA’ mitogenome. (**F**,**G**) The number distribution of DR with different groups and lengths in different mitogenomes.

In the comparative analysis conducted in this study, three types of repeat sequences were identified: simple sequence repeats (SSRs), LTRs, and DRs. The findings revealed that DRs were the most abundant in 10 *Malus* mitogenomes, followed by SSRs (Figure 4 and Table S6). By comparing the number of repetitions and repetition units, we can observe the diversity of SSRs within the *M. baccata* ‘ZA’ mitogenome (Figure 4A,B and Table S7). Specifically, a total of 115 SSRs were identified in the ‘ZA’ mitogenome, with tetra-nucleotide and mono-nucleotide types being predominant at 40 (34.7826%) and 39 (33.9130%), respectively (Figure 4C). Similarly, another sample from *M. baccata* exhibited a total of 121 SSRs, with tetra- (41) and mono-SSRs (41) also being the most abundant types observed. This trend was consistent across the other eight species as well (Table S6). Amongst all *Malus* mitogenomes analyzed, *M. hupehensis* var. mengshanensis displayed the highest number of SSRs at 125, while *M. domestica* (NC_018554) had the lowest count at 114; meanwhile, *M. domestica* ‘Yantai fuji 8’, *M. domestica* ‘Gala’, *M. domestica* ‘Honeycrisp’, and *M. sylvestris* all possessed 116 SSRs each. Finally, it is worth noting that no hexa-nucleotide repeat SSR was found among these 10 *Malus* mitogenomes.

Through the analysis of LTRs (Figure 4D and Table S8), it can be observed that both *M. baccata* ‘ZA’ and *M. baccata* exhibit a higher number (22) compared to the other eight *Malus* species (16, 17, and 18), except for *M. hupehensis* var. mengshanensis (23). Dispersed repeats can be categorized into four groups based on their match direction: forward/direct (F), reverse (R), complement (C), and palindromic (P). While each surveyed species possesses only one ‘R’ and one ‘C’, there are significant variations in the abundance of ‘F’ and ‘P’ elements they harbor (Figure 4E,F). For instance, *M. baccata* ‘ZA’ contains 181 ‘F’ repeats (43.614%) and 232 ‘P’ repeats (55.904%) (Figure 4E), whereas its counterpart in *M. baccata* reaches 245 and 249, respectively (Figure 4F). Furthermore, sequence lengths of DR predominantly range from 30 to 40 bp (Figure 4G and Table S9).

### 3.4. Codon Preference Analysis of Mitochondrial Coding Genes in M. baccata ‘ZA’

**Figure 5:**
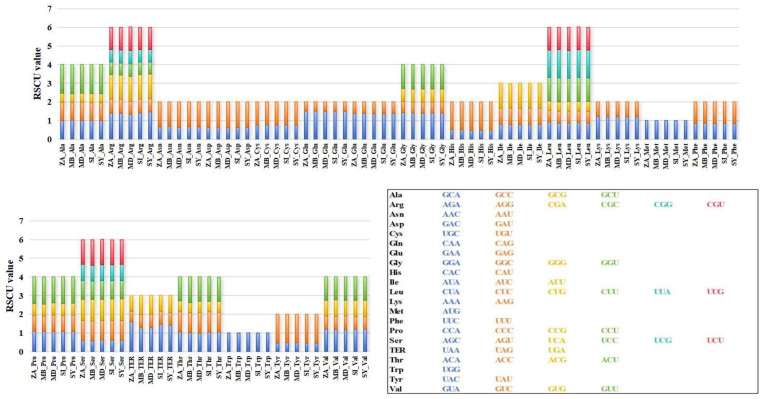
Relative synonymous codon usage of mitochondrial coding sequences in *M. baccata* ‘ZA’ and other four *Malus* species. Different codons encoding the same amino acid are distinguished by different colors. The codons corresponding to the six color blocks in the columnar stacking diagram are described in the box at the lower-right corner of the figure.

Codon usage bias is closely associated with the long-term evolution of species and can characterize the specificity of both species and genes. By comparing the RSCU values of mitochondrial coding sequences between *M. baccata* ‘ZA’ and four *Malus* species, it was observed that they exhibit consistent patterns in terms of codon type and frequency, as well as similar bias patterns (Figure 5). For *M. baccata* ‘ZA’ and other species, GCU codons are preferred for encoding Alanine (Ala), while Arginine (Arg) tends to utilize AGA and CGA types. Valine (Val) encoding favors GUA and GUU codons, whereas UAA is more commonly used as the stop codon (Figure 5). Additional calculations were performed to determine other characteristics related to codon usage, including four indexes: CAI, CBI, ENC, and FOP. The results presented in Table S10 indicate that *M. baccata* ‘ZA’ has the lowest CAI value among all analyzed samples at 0.166; however, both ‘ZA’ and *M. domestica* (NC_018554) display consistent CBI, ENC, and FOP values. In terms of GC3s statistics analysis, *M. baccata* exhibits the lowest value at 0.355, while *M. domestica* displays the highest value at 0.357 (Table S10).

### 3.5. Interspecific and Intraspecific Collinearity of Mitogenomes

**Figure 6:**
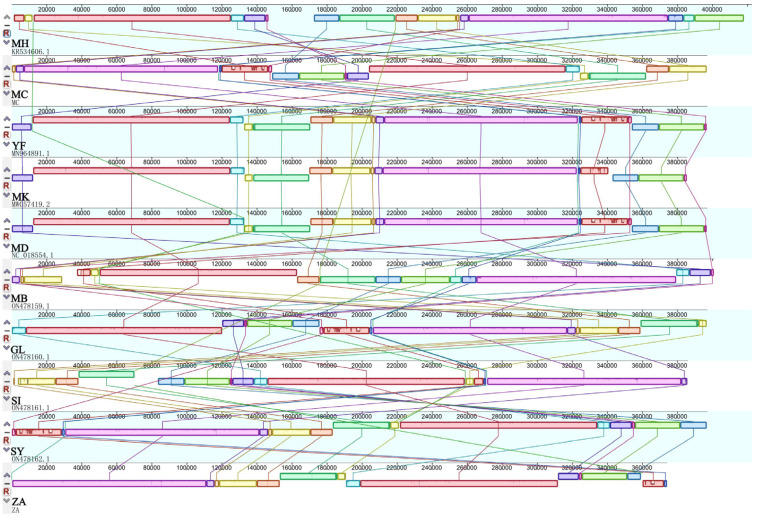
Interspecies collinearity comparison of *Malus* based on mitogenome. Different colors represent different collinear blocks, and the different species are connected by lines. For ease of representation, species names are reduced to two characters (see Figure 4).

Firstly, collinearity was detected in *M. baccata* ‘ZA’ using the BLAST algorithm (Figure S6A), revealing numerous local alignments within its mitogenome. A comparison with *M. baccata* in the database (NC_065224) showed smaller alignment blocks but confirmed the homology of the two mitogenomes (Figure S6B). Interestingly, some positions were reversed, indicating the change in direction of sequences within the mitogenome (Figure S6B). A further global comparison revealed a significant number of collinear blocks among all 10 *Malus* samples (Figure 6), including *M. baccata* ‘ZA’, while genome rearrangements (the order of collinear blocks is changed) were also common. For example, the connections around the larger purple and red blocks are more complex (Figure 6). As shown in Figure 6, molecular rearrangement led to a more dispersed distribution of collinear regions and hinted at instability and frequent recombination of *Malus* mitogenomes.

### 3.6. Assembly of Plastid Genome in M. baccata ‘ZA’ and Identification of MTPTs

**Figure 7:**
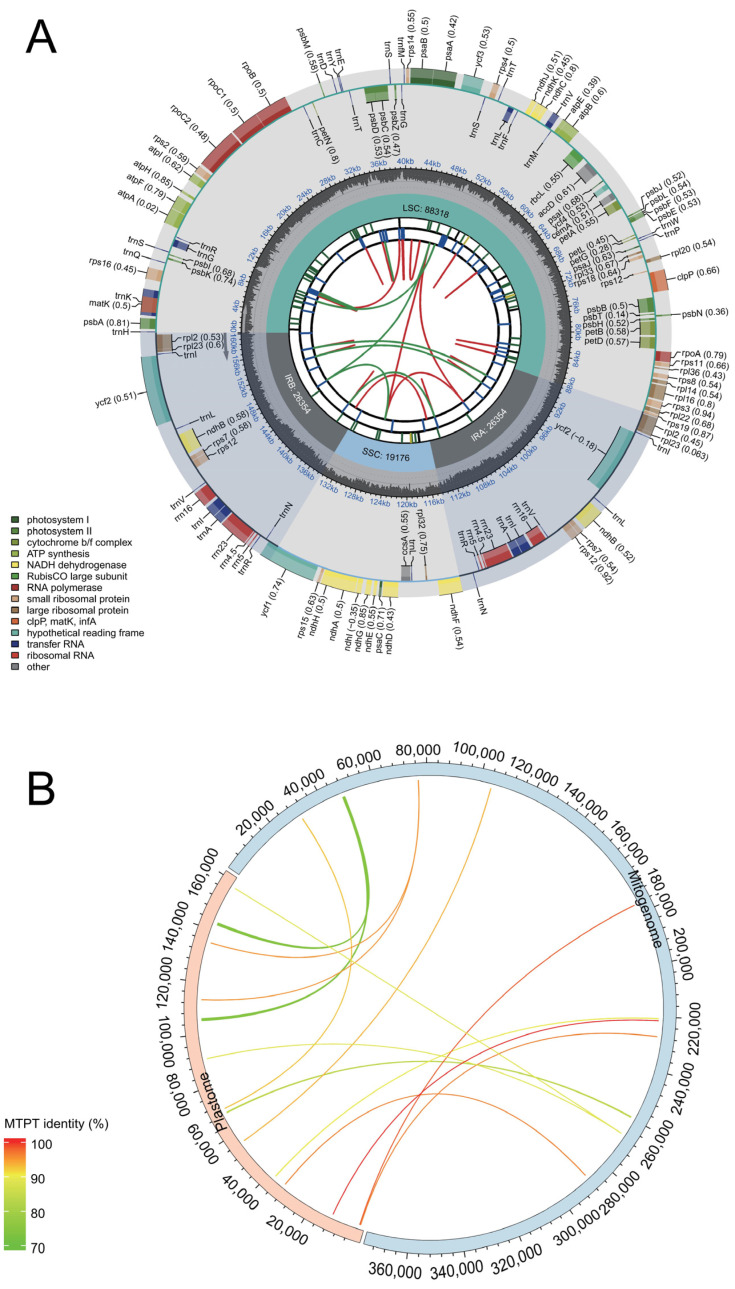
The overall features of chloroplast genome in *M. baccata* ‘ZA’ and MTPT transfer fragment analysis. (**A**) Gene classification and repeat sequence distribution in ‘ZA’ chloroplast genome. The genome map contains six layers of annotation information from the inside out, corresponding to dispersed repeats (D in red, P in green), long tandem repeats (colored blue), short tandem repeats (the seven types of microsatellite sequences are labeled as green_p1, yellow_p2, purple_p3, blue_p4, orange_p5, red_p6, and black_c), tetrad composition (LSC, SSC, IRa, and IRb), GC content, and gene name (codon usage bias is marked in parentheses), respectively. The lower-left corner of the map lists the color markers used by different functional genes. The gray arrows in the figure indicate the transcription direction of genes. (**B**) Transferred fragments from plastome to mitogenome in ‘ZA’. The red and blue half rings represent cpDNA and mtDNA, respectively. For both the mitogenome and the plastid genome, their direction is clockwise. The color of transferred fragments in the figure is determined according to the alignment results (BLAST identity).

The chloroplast genome (GenBank accession: OR876281) of *M. baccata* ‘ZA’ was successfully decoded using the same materials as those used for assembling the mitogenome. In this study, the complete plastid genome (*M. baccata* ‘ZA’) had a total length of 160,202 bp (base coverage = 4483.5; GC content: 36.5%). It consisted of a large single-copy region (LSC, 88,318 bp), a small single-copy region (SSC, 19,176 bp), and two inverted repeats (IRs, each spanning 26,354 bp) (Figure 7). In terms of sequence length, it accounted for approximately 42.83% of the total length of its mitogenome. Plastid genome annotation revealed a total of 129 genes, including 84 CDSs, 8 rRNAs, and 37 tRNAs (Figure 7A, Figures S7 and S8 and Table S11). Additionally, a significant number of repeat sequences (Figure 7A, Tables S12–S14) were identified in the cp genome of ‘ZA’, mainly including 50 DRs, 93 LTRs, and 71 SSRs.

The presence of intracellular DNA transfer leads to a significant number of foreign sequences in the mitogenome, including partial fragments derived from the nuclear and chloroplast genomes. Through homology analysis of the plastome and mitogenome, followed by manual filtering, a total of 14 instances of fragment transfer driven by plastids were identified in the *M. baccata* ‘ZA’ mitogenome (Figure 7B and Table 3). Furthermore, statistical analysis revealed that the respective proportions of transferred fragments in their corresponding genomes were 0.517% (mtDNA) and 1.835% (cpDNA). In all migration events (Table 3), sequence identity ranged from 73.933% (MTPT2) to 100% (MTPT14), with most being gene fragments rather than complete genes; the longest transfer reached an alignment length of 890 bp (Table 3).

### 3.7. Population Evolution Analysis Based on Mitochondrial Genome Polymorphisms in Malus

**Figure 8:**
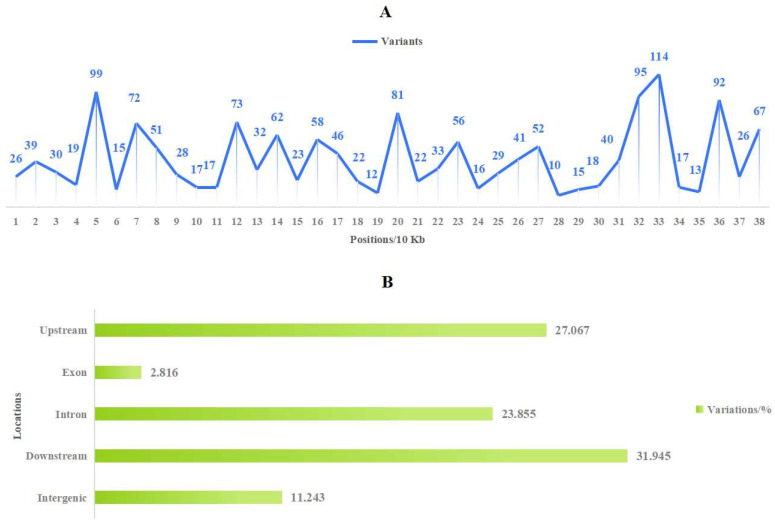
Distribution and type of *Malus* population variation based on mitogenome. (**A**) Distribution of high-quality variants in mitogenome (*M. baccata* ‘ZA’ mitogenome was set as the reference genome, and data were counted per 10 Kb). (**B**) The genomic region and type of population molecular variation.

**Figure 9:**
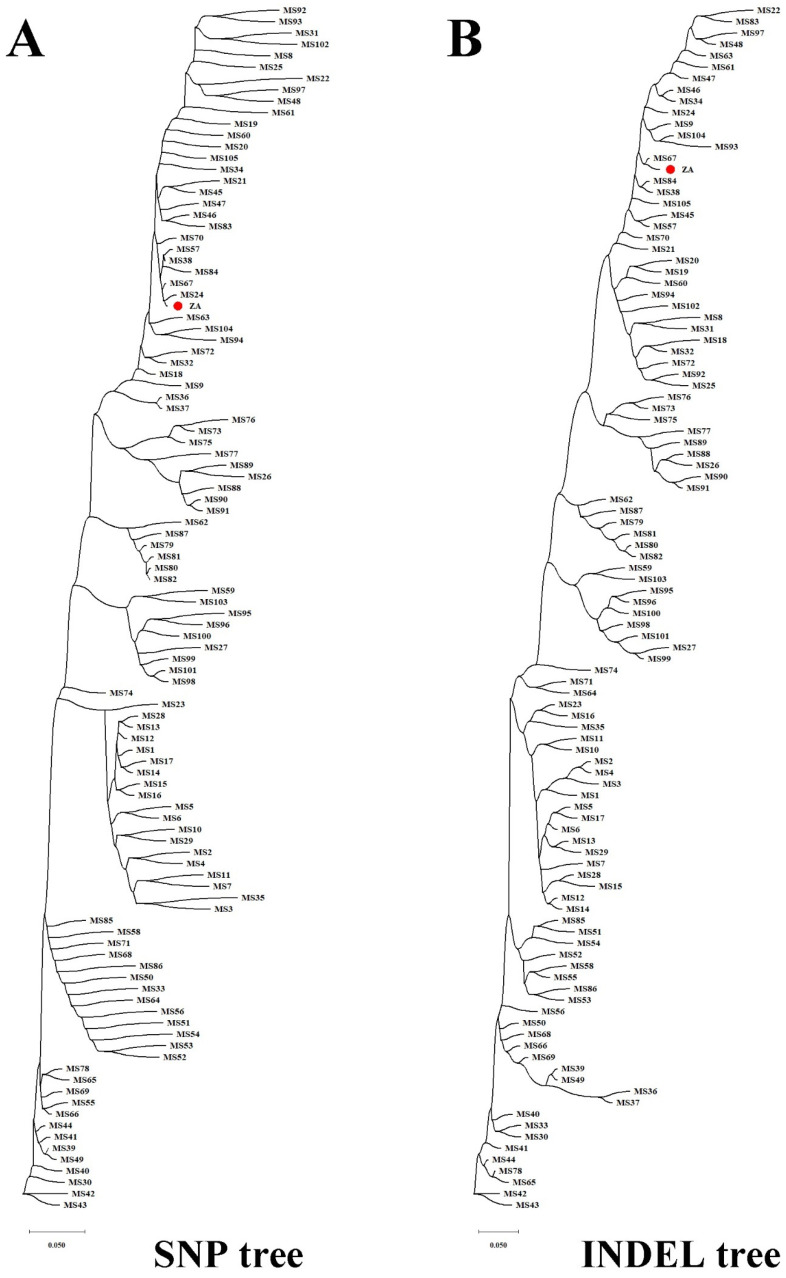
Population topology of 106 *Malus* germplasms based on molecular variations of mitogenome. (**A**) Single-nucleotide polymorphism tree. (**B**) Insertion/deletion tree. The biological location of *M. baccata* ‘ZA’ is highlighted in solid red circles (two trees constructed from filtered SNP/INDEL data), and the various details of other species are listed in Table S1. In phylogenetic analysis, the transformation of distance matrix to tree construction is calculated using the TaxAdd_BalME algorithm, and the corresponding scale represents the genetic distance.

The study of population genetics based on molecular variation holds theoretical significance in species identification and variety tracing. Firstly, utilizing the high-quality mitogenome (*M. baccata* ‘ZA’: PP826182) constructed in this study, we detected variations among different *Malus* species (Table S1). Subsequently, high-quality variations (1424 SNPs and 154 INDELs) were obtained by filtering missing rates and minor allele frequencies (Table S15). Notably, there were notable differences in the number of mutations at various positions within the mitogenome (Figure 8A) and more variants at 50, 70, 120, 200, 320, 330, 360, and 380 Kbp. Regarding SNP analysis based on these high-quality variations: base changes and transitions/transversions ratios (Ts/Tv) were calculated; and transversions (26,394) occurred more frequently than transitions (18,852), with a Ts/Tv value of 0.7143, as shown in Table S16. Additionally, when summarizing the key locations affected by these variations, it becomes evident that most occur within upstream regions, downstream regions, or introns of genes (Figure 8B). Furthermore, distance matrix calculations and phylogenetic tree construction allowed us to obtain topological relationships of ‘ZA’ and other 105 *Malus* individuals (Figure 9). In terms of the SNP tree (Figure 9A), *M. baccata* ‘ZA’, *M. domestica*, *M. baccata*, *M. sieversii*, *M. robusa*, and *M. prunifolia* are closely related, indicating a maternal inheritance relationship between them. Although some branches appear more dispersed in the INDEL tree, the same phenomenon exists (Figure 9B).

### 3.8. Phylogenetic Relationship between M. baccata ‘ZA’ and Other Species of Rosaceae

**Figure 10:**
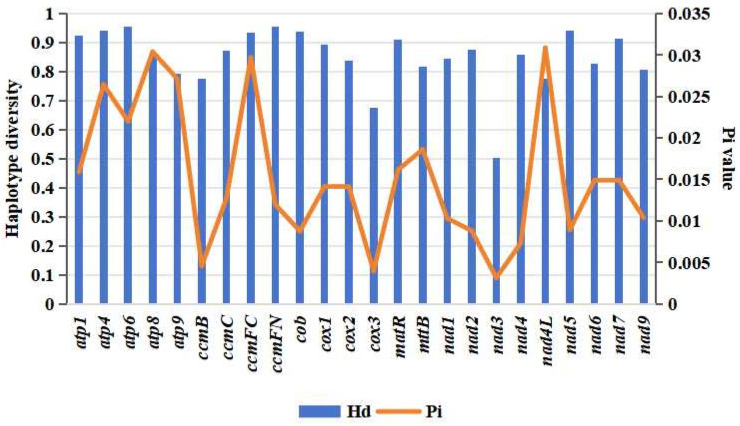
Haplotype diversity and nucleotide polymorphisms of 24 shared protein-coding genes in mitogenomes of 42 Rosaceae species.

The comparison of differentiation relationships in *Malus* using the mitogenome of *M. baccata* ‘ZA’ provides valuable insights into the cytoplasmic inheritance within the *Malus* genus. Subsequently, by conducting a comprehensive and extensive sample collection (NCBI RefSeq, Table 1 and Table S2), we described and characterized the evolutionary patterns of *M. baccata* ‘ZA’ within the Rosaceae family. Our analysis of 24 conserved and shared mtDNA coding genes from 42 species (belonging to 11 genera: *Malus*, *Sorbus*, *Rubus*, *Rosa*, *Pyrus*, *Prunus*, *Potentilla*, *Photinia*, *Geum*, *Fragaria,* and *Eriobotrya/Rhaphiolepis*) revealed nucleotide polymorphisms (π) ranging from 0.03087 (*nad4L*) to 0.00314 (*nad3*) (Figure 10), as well as varying levels of haplotype diversity, ranging from 0.502 (*nad3*) to 0.954 (*atp6* and *ccmFN*). These findings highlight significant differences and associations among these species (Figure 10).

**Figure 11:**
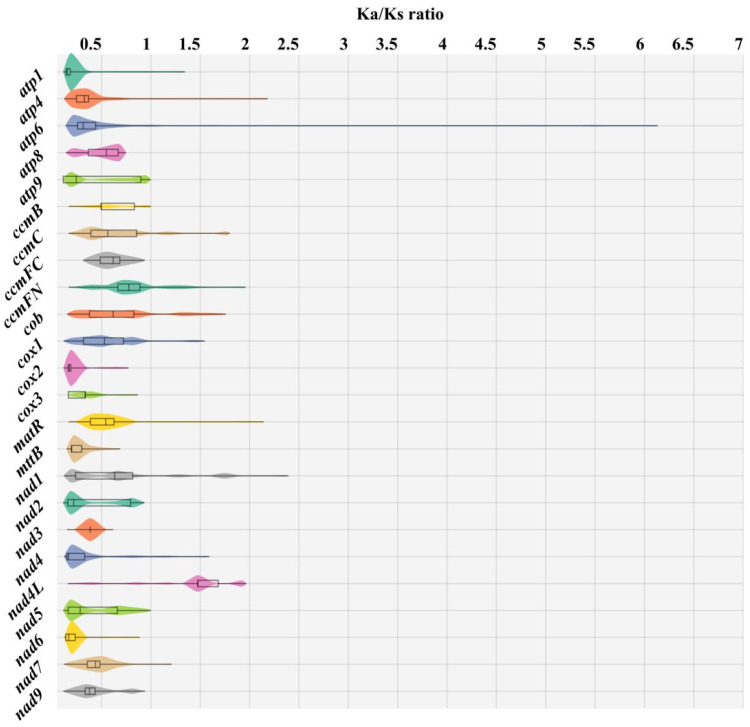
Distribution of Ka/Ks ratio of 24 mitochondrial genes in 42 Rosaceae species (including *M. baccata* ‘ZA’). The Ka/Ks values of single genes in different species pairs were calculated and counted respectively, and 0 and illegal values were treated as missing data. Different genes are represented by different colored violin graphs, where the width indicates how much of the data is distributed. In addition, extreme values, quartiles, and medians are indicated with box plots.

Furthermore, the selection pressure between gene pairs was individually calculated, revealing a substantial proportion of genes with a Ka/Ks ratio less than 1 (Figure 11). This indicates that these genes of 42 Rosaceae species (including *M. baccata* ‘ZA’) are subject to purifying selection. However, it is worth noting that the *nad4L* gene exhibited instances where Ka > Ks in certain species, suggesting its rapid evolution and positive selection (Figure 11).

**Figure 12:**
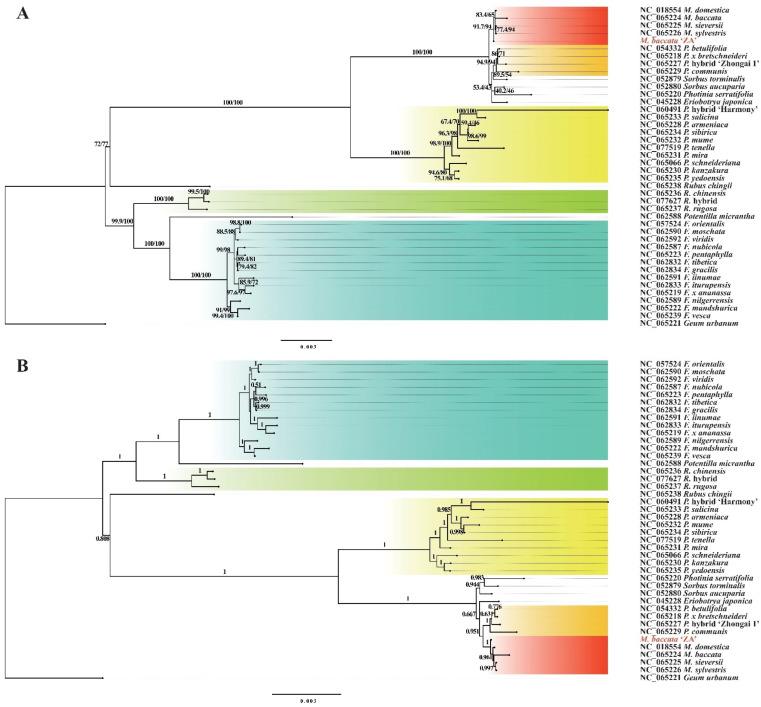
Phylogenetic topologies of *M. baccata* ‘ZA’ and other species of Rosaceae based on mitochondrial shared single-copy genes (23,838 nucleotide sites). (**A**) Maximum likelihood tree. (**B**) Bayesian inference tree. To distinguish, the location of ‘ZA’ in the topology is shown in bold red font. The outgroup of the unrooted tree was generated based on the first species in multiple sequence alignment (for this study, the tree was drawn at the outgroup *Geum urbanum*). The red, orange, yellow, green, and cyan blocks represent the *Malus*, *Pyrus*, *Prunus*, *Rosa,* and *Fragaria* genera, respectively. The numbers on the branches represent support, SH-aLRT support (%)/standard bootstrap percentage (%) for the ML tree, and the posterior probability density for the BI tree. The scale bar in the figure indicates the number of substitutions per site.

Based on the nucleotide variation loci mentioned above, we reconstructed the molecular evolutionary tree (ML and BI tree) of 42 species (including *M. baccata* ‘ZA’). As depicted in Figure 12A,B, both calculation methods yielded consistent branch structures, and the accuracy and reliability of the phylogenetic trees were confirmed through bootstrap percentage (BP) and posterior probability (PP) tests (Figure 12). In general, five genera, namely *Geum*, *Rubus*, *Rosa*, *Potentilla*, and *Fragaria*, formed a large evolutionary structure, while the remaining species constituted another main clade belonging to Amygdaloideae (Figure 12). Specifically, *Malus*, *Pyrus*, *Sorbus*, *Eriobotrya*, and *Photinia* were grouped together based on clustering relationships. Within the genus *Malus*, *M. baccata* ‘ZA’ formed a clade with *M. domestica*, *M. baccata*, *M. sieversii*, and *M. sylvestris* (Figure 12).

## 4. Discussion

Mitochondria are referred to as semi-autonomous organelles due to their limited genetic material and play crucial roles in energy metabolism in plant cells [59]. The complete genetic system of plants collectively constitutes the mitochondrial genome, chloroplast genome, and nuclear genome [60]. The mitochondrial DNA is influenced by the sequence of chloroplast or nuclear DNA through intracellular gene transfer [61]. Compared to the other two genomes, the plant mitogenome exhibits a lower evolutionary rate and has numerous applications in studying plant evolution, classification, and genetic diversity [49,62,63,64,65]. An analysis of mitogenome and genome-wide variation revealed convergent evolution during maize domestication and improvement [66]. By sequencing and assembling mitogenomes, researchers described the evolutionary relationships and adaptation strategies of four *Hevea* species [67]. To assess population structure and variation in Asian rice and wild rice, statistical values such as fixation index (Fst) were calculated using mitogenome data. The results suggested that *indica* rice may have a significant genetic distance from *japonica* rice [49]. However, due to the complexity of structural variations and transfer fragments within plant mitogenomes, assembly remains a challenging task [61,68]. For the *Malus* genus, there are approximately ten records of mitogenomes available in NCBI encompassing only seven species, which significantly limits research progress on *Malus* speciation.

Due to extensive outcrossing and natural mutation of *Malus* species, the resulting hybrids and mutants not only expand their ecological range and genetic diversity but also pose challenges for species traceability and germplasm identification [10,69]. For instance, *M. baccata* ‘ZA’ (a dwarf mutant) serves as a clear example. Initially, ‘ZA’ was reported as a mutant type of *M. baccata*. Morphological and physiological comparisons in this study confirmed that the plant height and leaf shape of ‘ZA’ were significantly changed compared with WT (Figure 1). Despite its mention in previous studies, there is limited research on the taxonomic and genetic aspects of ‘ZA’. In a study investigating the origin of cultivated apples, SNPs were identified through integrating resequencing and transcriptome data, including that of *Malus baccata* ‘ZA’. Population structure analysis and gene flow assessment revealed distinct ancestors for Chinese and European cultivated apples, with contributions from *M. baccata* and *M. hupehensis* through gene introgressions [7]. Through differential expression gene (DEG) annotation and hormone assay, it was speculated that the down-regulation of the *MbIAA19* gene in ‘ZA’ plays a crucial role in plant dwarfing and auxin regulation—a conclusion confirmed by subsequent genetic transformation experiments [70]. However, despite these references to ‘ZA’, its maternal origin remains unknown, along with evolutionary clues. To understand this issue comprehensively, we decoded the complete mitogenome of ‘ZA’ using high-throughput sequencing while describing its organelle inheritance as well as variation pattern. The reference sequence length of the ‘ZA’ mitogenome was 374,023 bp (Figure 3 and Table 1), which differed from published *Malus* species (385~423 Kb) (Table 1). Our results identified a total of 54 genes, including 24 core protein-coding genes that were similar to other *Malus* species [53,54] (Figure 3, Table 2 and Table S4). Despite conserved coding genes across different mitogenomes, inconsistent gene arrangement is common due to structural and sequence differences. Although we found numerous collinear blocks in sequence homology comparison, genome rearrangement events in 10 *Malus* plants still require attention [18] (Figure 6 and Figure S6). As reported in *Fragaria* [18], the authors used mitochondrial genome data from 13 species to identify potential genome rearrangement events and found large-scale structural variations. The relative synonymous codon usage index provides insight into usage patterns. Codon usage analysis revealed amino acid preferences for Ala, Arg, and Val in ‘ZA’ mitogenome PCGs with TAA as the frequent stop codon occurrence, similar to *Punica granatum* and *Camellia sinensis* studies [16,71] (Figure 5). Repetitive sequences play a crucial role as significant indicators of mitogenome evolution, and investigating their quantitative differences across different species is instrumental in uncovering deeper genomic variation information. In *Sorghum* mitogenomes [17], A/T, AC/GT, AG/CT, and AT/AT motifs were identified as different types of SSRs, and A/T was the most abundant category. Similarly, this situation also exists in the analysis results of ‘ZA’ in this paper. MTPT transfer DNA reflects the exchange of genetic material between organelles. In this study, highly similar segments were identified in the ‘ZA’ cp genome (Figure 7 and Table 3), which constituted 0.517% of the mitogenome. Comparable findings were observed in other species, with percentages of 1.56% (*Camellia Duntsa*), 0.54% (*Punica granatum*), and 2.10% (*Ilex metabaptista*) [16,71,72]. Population genetic analysis revealed low nucleotide diversity among mitochondrial coding genes in the compared Rosaceae species (Figure 10), with most genes showing no evidence of positive selection during evolution (Figure 11). Taken together, these data indicate a high level of conservation in mitochondrial genes across ‘ZA’ and different *Malus* species, including both cultivated and wild varieties, as evidenced by gene count, codon usage, variation sites, and selection pressure metrics. However, further exploration is needed to understand the complexity of repeat sequences and transfer fragments responsible for high polymorphism and structural variations within non-coding regions [53].

For a considerable duration, the interspecific status and species classification of *M. baccata* have garnered significant attention. Apart from *M. baccata* ‘ZA’ mentioned in this article, various forms of *M. baccata* (e.g., *M. baccata* f. *gracilis*, var. *latifolia*, f. *villosa*) and geographically diverse individuals serve as representatives within this category. By reconstructing the evolutionary relationships among chloroplast genomes of different *Malus* species, it was observed that *M. baccata* f. *gracilis* clustered together with four other species (*M. hupehenisis*, *M. sikkimensis*, *M. toringoides*, and *M. rokii*) [9]. Based on the genomic assembly of a sample from Shanxi province in China, approximately 47.56% of the genes in *M. baccata* exhibited a one-to-one orthology relationship with those found in the genome of *M. domestica* [73]. Through SSR amplification and Fst calculation involving 391 *Malus* accessions, it was determined that both *M. baccata* and *M. × robusta* displayed greater similarity to DomSoviet (*M. domestica* originating from former Soviet regions) while exhibiting more distant genetic relatedness to Chinese and Western varieties of domesticated apples [74]. In an analysis conducted on twelve individuals of *M. baccata* [3], both the maximum likelihood tree and the Bayesian inference tree revealed two primary branches within the phylogenetic structure of this species. In this study, the maternal genetic characteristics of *M. baccata* ‘ZA’ were found to be influenced by *M. baccata*, *M. sieversii*, and other closely related species (Figure 9 and Figure 12), which has significantly enhanced our understanding of molecular genetics in both *M. baccata* and Rosaceae. These examples clearly demonstrate that in the era of extensive systematic evolutionary research facilitated by big data [20,21,22,23], relying solely on partial data is insufficient for comprehensive analysis. Therefore, it is imperative to provide additional reference sequences and molecular datasets to enable accurate inference regarding complex interspecies relationships within *Malus*.

However, there are still some limitations in the research content of this paper. For instance, the single master circle model fails to fully and accurately depict the diverse and dynamic structural information of mitogenomes [17,75]. Fortunately, advancements in sequencing technology (PacBio high-fidelity reads, HiFi) and assembly tools (graph-based sequence assembly toolkit, GSAT; plant mitogenome assembly toolkit, PMAT) will aid us in enhancing our experimental methods and resolving these challenges in the future [75,76]. Moreover, the development of the ptGAULprocess serves as a reference case for improving continuity and accuracy in chloroplast genome studies [77]. Furthermore, the release and publication of the *M. baccata* ‘ZA’ mitogenome offers novel insights into complex evolutionary relationships within *Malus* and even Rosaceae. To some extent, it also establishes a theoretical foundation for enhancing varieties and utilizing production materials—particularly valuable wild germplasms.

## 5. Conclusions

The complete mitogenome of *M. baccata* ‘ZA’ was decoded and obtained through high-throughput sequencing and assembly methods in this study. Detailed comparative genomics analysis characterized the similarities and differences in the mitogenomes of *Malus*, including genome GC content, number of core genes, distribution of repeat sequences, and relative synonymous codon usage. In the mitogenomes of *M. baccata* ‘ZA’ and *M. baccata*, homology blocks are widely distributed, while there are similar regions within the ‘ZA’ mitogenome and between the mitogenome and plastome of ‘ZA’. Furthermore, clear rearrangement events were observed in the mitogenomes of *Malus*. By mapping the evolutionary position of *M. baccata* ‘ZA’ within *Malus* and Rosaceae based on rich interspecific variation and relatively conserved core genes in mitogenomes, this study contributes to future germplasm identification and conservation efforts.

## Figures and Tables

**Table 1:** Comparison of mitogenomes assembled in this study with published sequences of *Malus*.

Family and Genus	Species	Reference	GenBank Accession	Sequence Length (bp)	Molecular Type	GC Content (%)	GC Skew
Rosaceae, Malus	*M. baccata* ‘ZA’	This study	PP826182	374,023	Circular DNA	45.4	−0.2695~0.2706
	*M. baccata*	[53]	NC_065224 ^1^	400,769	Circular DNA	45.4	−0.2739~0.2682
	*M. domestica*	[54]	NC_018554 ^1^	396,947	Circular DNA	45.4	−0.2717~0.2695
	*M. domestica* ‘Yantai fuji 8’	[55]	MN964891	396,947	Circular DNA	45.4	−0.2717~0.2695
	*M. domestica* ‘Gala’	[53]	ON478160	396,946	Circular DNA	45.4	−0.2695~0.2717
	*M. domestica*	*	OX352770	400,843	Linear DNA	45.4	—
	*M. domestica*	*	OX352778	392,471	Linear DNA	45.4	—
	*M. domestica*	*	OX352780	400,843	Linear DNA	45.4	—
	*M. domestica*	*	OX352782	400,843	Linear DNA	45.4	—
	*M. domestica* ‘Honeycrisp’	This study; Data source: [32]	OR876282	396,949	Circular DNA	45.4	−0.2717~0.2695
	*M. domestica* ‘Fuji’	[56]	—	436,177	—	45.4	—
	*M. hupehensis* var. mengshanensis	[57]	KR534606	422,555	Circular DNA	45.2	−0.2682~0.2723
	*M. kansuensis*	[58]	MW057419	385,436	Circular DNA	45.3	−0.2717~0.2711
	*M. sieversii*	[53]	NC_065225 ^1^	385,869	Circular DNA	45.4	−0.2711~0.2692
	*M. sylvestris*	[53]	NC_065226 ^1^	396,940	Circular DNA	45.4	−0.2711~0.2692
	*M. sylvestris*	*	OX352768	423,217	Linear DNA	45.5	—
	*M.* × *robusta*	*	OY720342	385,872	Linear DNA	45.4	—
	*M.* ‘SH6’	[56]	—	453,068	—	45.0	—
	*M.* ‘Flame’	[56]	—	441,454	—	45.3	—
	*M.* ‘Royalty’	[56]	—	397,430	—	45.3	—

Note: The asterisk position (*) indicates that it was mentioned in the Wellcome Sanger Tree of Life Programme. The accession number marked numerically in the upper right corner (^1^) is the NCBI reference sequence.

**Table 2:** Annotated genes in the mitochondrial genome of *M. baccata* ‘ZA’.

Gene Category	Gene Function	Gene Name
Core protein-coding genes	Subunit of NADH dehydrogenase (complex I)	*nad1* ^c^, *nad2* ^c^, *nad3*, *nad4* ^b^, *nad4L*, *nad5* ^c^, *nad6*, *nad7* ^c^, *nad9*
	Apocytochrome b (complex III)	*cob*
	Subunit of cytochrome c oxidase (complex IV)	*cox1*, *cox2*, *cox3*
	Subunit of ATP synthase (complex V)	*atp1*, *atp4*, *atp6*, *atp8*, *atp9*
	Cytochrome c biogenesis	*ccmB*, *ccmC*, *ccmFC* ^a^, *ccmFN*
	Maturase	*matR*
	Transport membrane protein	*mttB*
Variable PCGs	Large subunit of ribosome	*rpl5*, *rpl10*, *rpl16*
	Small subunit of ribosome	*rps1*, *rps3*, *rps4*, *rps12*, *rps13*, *rps14*
	Subunit of succinate dehydrogenase (complex II)	*sdh3*, *sdh4* ^d^
tRNA genes	Transfer RNA	*trnC-GCA*, *trnD-GUC*, *trnE-UUC* ^a,d^, *trnF-GAA* ^e^, *trnG-GCC*, *trnH-GUG*, *trnI-CAU*, *trnK-UUU*, *trnM-CAU* ^a,d^, *trnfM-CAU*, *trnN-GUU*, *trnP-UGG* ^d^, *trnQ-UUG*, *trnS-UGA*, *trnW-CCA*, *trnY-GUA*
rRNA genes	Ribosomal RNA	*rrn5*, *rrn18*, *rrn26*

Note: Genes with multiple introns, or copies, are indicated with a lowercase letter, where ^a^ is one intron, ^b^ is three introns, ^c^ is four introns, ^d^ is two copies, and ^e^ is three copies.

**Table 3:** The identification of MTPT transfer fragments in *M. baccata* ‘ZA’.

MTPT Transfer Fragment	MTDNA Locations	CPDNA Locations	Identity (%)	Alignment Length (bp)	Mismatches	Gap Openings	Expected Value	Bit Score	Sequence Annotation
1	47,398…48,244	106,040…105,189	74.032	878	171	44	5.21 × 10^−82^	305	Partial *rrn16*
2	47,386…48,244	142,469…143,332	73.933	890	175	44	5.21 × 10^−82^	305	Partial *rrn16*
3	259,462…259,783	68,689…68,375	83.333	324	43	5	5.25 × 10^−77^	289	Partial *psbE*, Partial (*psbE_petL*)
4	220,541…220,679	37,818…37,682	90	140	10	4	1.19 × 10^−43^	178	Partial *psbC*
5	30,188…30,295	70,201…70,310	92.727	110	6	2	1.55 × 10^−37^	158	Partial (*petG_trnW-CCA*), complete (*trnW-CCA*), Partial (*trnW-CCA_trnP-UGG*)
6	227,435…227,519	35…118	96.471	85	2	1	5.60 × 10^−32^	139	Partial (*rpl2_trnH-GUG*), complete *trnH-GUG*, Partial (*trnH-GUG_psbA*)
7	287,676…287,759	32,888…32,971	96.429	84	3	0	5.60 × 10^−32^	139	Partial (*psbM_trnD-GUC*), complete *trnD-GUC*, Partial (*trnD-GUC_trnY_GUA*)
8	76,742…76,826	113,280…113,196	95.349	86	2	2	7.24 × 10^−31^	135	Partial (*trnR-ACG_trnN-GUU*), complete *trnN-GUU*, Partial (*trnN-GUU_ndhF*)
9	76,742…76,826	135,241…135,325	95.349	86	2	2	7.24 × 10^−31^	135	Partial (*ycf1_trnN-GUU*), complete *trnN-GUU*, Partial (*trnN-GUU_trnR-ACG*)
10	104,344…104,422	55,944…55,866	93.671	79	5	0	7.29 × 10^−26^	119	Partial (*trnV-UAC_trnM-CAU*), complete *trnM-CAU*, Partial (*trnM-CAU_atpE*)
11	176,322…176,385	395…458	98.438	64	1	0	3.39 × 10^−24^	113	Partial *psbA*
12	266,723…266,801	90,558…90,484	88.608	79	5	3	4.42 × 10^−18^	93.5	Partial (*rpl23_trnI-CAU*), complete *trnI-CAU*
13	266,723…266,801	157,963…158,037	88.608	79	5	3	4.42 × 10^−18^	93.5	Complete *trnI-CAU*, Partial (*trnI-CAU_rpl23*)
14	221,409…221,439	11,357…11,387	100	31	0	0	1.61× 10^−7^	58.4	Partial *atpA*

## Data Availability

The assembled sequences and annotations of this study have been uploaded to the NCBI public database (GenBank), numbered PP826182 (mitochondrial genome of *M. baccata* ‘ZA’), OR876281 (chloroplast genome of *M. baccata* ‘ZA’), and OR876282 (*M. domestica* ‘Honeycrisp’ mitogenome). At the same time, Illumina data of next-generation sequencing related to the mitogenome assembly of *M. baccata* ‘ZA’ have been submitted to China National Center for Bioinformation (BIG Submission), and the corresponding accession numbers are BioProject: PRJCA025477 and Genome Sequence Archive (GSA): CRA016093, CRR1120135. In addition, other relevant content and information can be obtained by contacting the corresponding authors.

## Supplementary Materials

The following supporting information can be downloaded at: https://www.mdpi.com/article/10.3390/biom14080912/s1, Figure S1: Unicycler assembly process of ‘ZA’ mitogenome; Figure S2: Coverage depth histogram of clean reads for *M. baccata* ‘ZA’ mitogenome; Figure S3. The differences and similarities of GC content/skew between 10 *Malus* mitogenomes; Figure S4: The location and structure of *cis*-splicing genes in *M. baccata* ‘ZA’ mitogenome; Figure S5: Trans-splicing genes (*nad1*, *nad2*, and *nad5*) in *M. baccata* ‘ZA’ mitogenome; Figure S6: Similarity map based on global alignment of mitogenomes in *M. baccata* and *M. baccata* ‘ZA’; Figure S7: *Cis*-splicing genes (exon and intron composition) in the cp genome of *M. baccata* ‘ZA’; Figure S8: Distribution and position of *rps12* (*trans*-splicing gene) in chloroplast genome of *M. baccata* ‘ZA’; Table S1: Sample information and sequencing statistics of 105 *Malus* spp. Resources; Table S2: Mitogenomes of Rosaceae involved in this study (except *Malus*); Table S3: Summary of coverage of mitogenome assembly in *M. baccata* ‘ZA’; Table S4: Annotation information of predicted protein-coding genes in the *M. baccata* ‘ZA’ mitogenome; Table S5: Eight intron-contained mitochondrial genes in *M. baccata* ‘ZA’ mitogenome; Table S6: Identified SSRs in mitogenomes of *M. baccata* ‘ZA’ and others; Table S7: Different type SSRs in mitogenome of *M. baccata* ‘ZA’; Table S8: LTR distribution in *M. baccata* ‘ZA’ mitogenome; Table S9: The prediction of DRs in *M. baccata* ‘ZA’ mitogenome; Table S10: Codon usage patterns of mitochondrial protein-coding sequences in five *Malus* species; Table S11: Annotated protein-coding genes in *M. baccata* ‘ZA’ cp genome; Table S12: Dispersed repeats classification (P/D) and position in *M. baccata* ‘ZA’ cp genome; Table S13: Identified long tandem repeats in *M. baccata* ‘ZA’ cp genome; Table S14: Frequency of identified SSR motifs in cp genome of *M. baccata* ‘ZA’; Table S15: Population variation statistics of 106 *Malus* species based on mitogenomes; Table S16: Mitogenome SNP variations in 106 *Malus* species.

## Abbreviations

BI: Bayesian inference; BLAST, basic local alignment search tool; bp, base pairs; BP, bootstrap percentage; CAI, codon adaptation index; CBI, codon bias index; CDS, coding sequence; CLR, continuous long reads; cp, chloroplast; CPGView, Chloroplast Genome Viewer; CTAB, cetyl trimethyl ammonium bromide; DEG, differential expression gene; DRs, dispersed repeats; ENC, effective number of codons; FOP, frequency of optimal codons; Fst, Wright’s F-statistics/fixation index; Gb, gigabase; GSAT, graph-based sequence assembly toolkit; HiFi, high-fidelity reads; INDELs, insertions and deletions; IPMGA, intelligent plant mitochondrial genome annotator; IRs, inverted repeats; Ka, nonsynonymous substitution rates; Ks, synonymous substitution rates; LSC, large single-copy region; LTRs, long tandem repeats; MEGA, molecular evolutionary genetics analysis; ML, maximum likelihood; mt, mitogenome; mtDNA, mitochondrial DNA; MTPTs, mitochondrial plastid DNAs; PCGs, protein-coding genes; PE, paired-end reads; PMAT, plant mitogenome assembly toolkit; PP, posterior probability; pt, plastome; rRNAs, ribosomal RNAs; RSCU, relative synonymous codon usage; SNPs, single nucleotide polymorphisms; spp., species; SSC, small single-copy region; SSRs, simple sequence repeats; STRs, short tandem repeats; tRNAs, transfer RNAs; WT, wild type.
