# Supplementary material for: Morphological Structure Identification, Comparative Mitochondrial Genomics and Population Genetic Analysis toward Exploring Interspecific Variations and Phylogenetic Implications of Malus baccata ‘ZA’ and Other Species
Source: Biomolecules. 2024 Jul 26;14(8):912. doi: 10.3390/biom14080912 (PMC11352593; doi:10.3390/biom14080912)
Supplement: Supplementary file 1 [file biomolecules-14-00912-s001.zip › biomolecules-3095696-supplementary.pdf]

Table S1. Sample information and sequencing statistics of 105 *Malus* spp. resources.

Sample number	Species classification	Cultivar name	F bases	R bases
MS1	*M. domestica*	'Hanfu'	2,871,591,897	2,870,965,066
MS2	*M. domestica*	'Liangxiang'	2,542,181,653	2,541,703,144
MS3	*M. domestica*	'Pinklady'	2,510,997,000	2,510,997,000
MS4	*M. sylvestris*		2,793,692,032	2,793,303,926
MS5	*M. domestica*	'Huahong'	2,586,984,670	2,586,663,382
MS6	*M. domestica*	'Sinanogold'	2,632,346,082	2,631,992,140
MS7	*M. domestica*	'Cameo'	2,880,289,076	2,879,981,717
MS8	*M. domestica*		2,749,614,802	2,749,077,772
MS9	*M. domestica*	'Binzi'	2,482,862,550	2,482,862,550
MS10	*M. domestica*	'Xiajia'	2,951,051,012	2,950,581,168
MS11	*M. domestica*	'Dahongrong'	2,627,711,250	2,627,711,250
MS12	*M. domestica*	'Alps-otome'	2,696,472,643	2,696,119,893
MS13	*M. sp.*	'SH40'	2,489,255,700	2,489,255,700
MS14	*M. domestica*	'Hualihu'	2,783,611,350	2,783,611,350
MS15	*M. sp.*	'M'	3,766,432,950	3,766,432,950
MS16	*M. sp.*	'MM106'	3,171,938,550	3,171,938,550
MS17	*M. domestica*	'Liaofu'	3,148,514,776	3,147,799,671
MS18	*M. micromalus*		2,513,563,019	2,513,101,194
MS19	*M. prattii*		2,395,724,760	2,395,349,531
MS20	*M. robusta*		3,077,424,750	3,077,424,750
MS21	*M. domestica*		2,276,232,495	2,275,838,789
MS22	*M. domestica*	'Yili'	2,906,829,861	2,906,374,633
MS23	*M. sp.*	'Flame'	3,316,635,269	3,316,186,691
MS24	*M. sp.*	'128'	2,514,159,450	2,514,159,450
MS25	*M. domestica*	'Yuling'	2,438,057,250	2,438,057,250
MS26	*M. domestica*	'Guifuren'	2,427,366,589	2,426,931,234
MS27	*M. hupehensis*		2,817,841,192	2,817,433,584
MS28	*M. sp.*	'M7'	3,177,714,000	3,177,714,000
MS29	*M. domestica*		2,438,128,800	2,438,128,800
MS30	*M. domestica*		2,488,520,850	2,488,520,850
MS31	*M. sikkimensis*		2,723,538,318	2,722,910,721
MS32	*M. domestica*	'Hongmanao'	2,839,326,150	2,839,326,150
MS33	*M. domestica*	'Jiguan'	3,023,496,814	3,023,007,000
MS34	*M. domestica*	'Longfeng'	2,779,342,532	2,778,671,403
MS35	*M. orientalis*		2,570,902,937	2,570,371,607
MS36	*M. sp.*	'Robinson'	3,669,677,400	3,669,677,400
MS37	*M. sp.*	'Mark'	4,871,723,250	4,871,723,250
MS38	*M. sp.*	'M27'	3,170,529,150	3,170,529,150
MS39	*M. sp.*	'B150'	3,066,078,750	3,066,078,750
MS40	*M. sp.*	'B396'	2,650,283,100	2,650,283,100
MS41	*M. sp.*	'V1'	3,662,740,350	3,662,740,350

MS42	*M. sp.*	'V6'	2,811,612,600	2,811,612,600
MS43	*M. sp.*	'B9'	2,469,765,333	2,465,390,337
MS44	*M. sp.*	'Bp'	2,145,712,200	2,145,712,200
MS45	*M. sieversii*	'Ha'	2,664,897,896	2,664,528,489
MS46	*M. domestica*	'Lh'	3,384,200,567	3,383,332,685
MS47	*M. sieversii*	'Xinjiang'	3,196,967,085	3,196,420,505
MS48	*M. sieversii*	'Yili'	2,231,740,523	2,231,418,699
MS49	*M. domestica*	'Dx'	5,062,107,600	5,062,107,600
MS50	*M. fusca*		2,804,546,942	2,804,184,004
MS51	*M. sieversii*	'Hongrou'	3,106,286,642	3,105,654,215
MS52	*M. sieversii*		2,698,465,695	2,698,284,452
MS53	*M. sieversii*	'kirghisorum'	2,808,164,961	2,807,545,236
MS54	*M. turkmenorum*		2,679,672,212	2,679,090,939
MS55	*M. sp.*	'Lj'	2,487,834,548	2,486,973,264
MS56	*M. sieversii*		2,589,814,382	2,589,326,452
MS57	*M. sp.*	'Cz'	2,186,514,750	2,186,514,750
MS58	*M. asiatica*		3,419,591,595	3,418,572,935
MS59	*M. asiatica*		3,308,445,950	3,308,146,913
MS60	*M. micromalus*	'Haihong'	3,160,269,120	3,159,573,095
MS61	*M. halliana*		2,662,910,392	2,662,402,663
MS62	*M. halliana*		2,884,561,650	2,884,561,650
MS63	*M. prunifolia*	'Laiwu'	2,489,245,826	2,488,941,222
MS64	*M. prunifolia*	'laoshan'	3,068,472,900	3,068,472,900
MS65	*M. prunifolia*	'Nb'	2,485,050,600	2,485,050,600
MS66	*M. prunifolia*	'Ny'	2,747,247,000	2,747,247,000
MS67	*M. prunifolia*	'Laiyang'	2,300,958,600	2,300,958,600
MS68	*M. prunifolia*	'Lengunzi'	2,570,084,550	2,570,084,550
MS69	*M. prunifolia*	'Regunzi'	2,435,760,600	2,435,760,600
MS70	*M. prunifolia*	'Xinzhuang'	2,064,091,650	2,064,091,650
MS71	*M. sp.*	'Kelsey'	2,860,942,112	2,860,418,123
MS72	*M. manshurica*		2,843,824,965	2,843,289,469
MS73	*M. sp.*	'Brandywine'	2,393,068,800	2,393,068,800
MS74	*M. yunnanensis*		3,775,820,100	3,775,820,100
MS75	*M. sp.*	'Prairie-rose'	3,082,727,400	3,082,727,400
MS76	*M. ioensis*		2,786,061,912	2,785,490,133
MS77	*M. tschonoskii*		2,686,211,554	2,685,801,522
MS78	*M. sp.*	'Radiant'	2,826,894,060	2,826,423,463
MS79	*M. sp.*	'Erb'	2,960,890,650	2,960,890,650
MS80	*M. sp.*	'Fed'	2,839,386,900	2,839,386,900
MS81	*M. sp.*	'A560'	2,519,833,650	2,519,833,650
MS82	*M. sp.*	'129'	2,401,658,700	2,401,658,700
MS83	*M. baccata*	'Neimenggu'	2,683,349,850	2,683,349,850
MS84	*M. sp.*	'Pinkspire'	3,834,662,907	3,833,948,789
MS85	*M. sp.*	'Strawberry-parfait''	3,234,919,950	3,234,919,950

MS86	*M. sp.*	'Sparkler'	2,702,619,618	2,702,253,258
MS87	*M. floribunda*		2,762,084,657	2,761,212,943
MS88	*M. toringoides*		3,287,389,950	3,287,389,950
MS89	*M. xiaojinensis*		2,963,267,756	2,962,870,979
MS90	*M. toringoides*	'1'	2,413,209,750	2,413,209,750
MS91	*M. toringoides*	'2'	3,031,642,050	3,031,642,050
MS92	*M. baccata*	'Manzhouli'	2,700,005,832	2,699,528,470
MS93	*M. baccata*	'Manzhouli1'	2,700,557,700	2,700,557,700
MS94	*M. sieboldii*		2,841,616,433	2,841,868,378
MS95	*M. sp.*	'Lingyungong'	3,126,219,861	3,125,670,304
MS96	*M. sargentii*		2,618,441,240	2,617,704,241
MS97	*M. sp.*	'Baotou'	2,979,770,100	2,979,770,100
MS98	*M. sp.*	'Laoshan'	2,049,001,050	2,049,001,050
MS99	*M. baccata*	'Hebei1'	2,315,593,050	2,315,593,050
MS100	*M. sp.*	'Shanxi'	2,472,012,716	2,471,586,103
MS101	*M. baccata*	'Zhongzhen'	2,347,576,501	2,347,105,678
MS102	*M. baccata*	'Weizhi'	2,271,803,700	2,271,803,700
MS103	*M. baccata*	'Gangsu'	2,354,259,600	2,354,259,600
MS104	*M. baccata*	'Shandong'	2,595,714,030	2,594,995,975
MS105	*M. baccata*	'Hebei2'	2,361,297,437	2,360,744,866

Table S2. Mitogenomes of Rosaceae involved in this study (*except Malus*).

RefSeq id	Organism name	Mitogenome type	Mitogenome size/bp
NC_045228.1	*Eriobotrya japonica*	Circular	434,980
NC_062834.1	*Fragaria gracilis*	Circular	283,031
NC_062591.1	*Fragaria iinumae*	Circular	328,819
NC_062833.1	*Fragaria iturupensis*	Circular	351,183
NC_065222.1	*Fragaria mandshurica*	Circular	313,022
NC_062590.1	*Fragaria moschata*	Circular	277,672
NC_062589.1	*Fragaria nilgerrensis*	Circular	315,211
NC_062587.1	*Fragaria nubicola*	Circular	299,474
NC_057524.1	*Fragaria orientalis*	Circular	275,143
NC_065223.1	*Fragaria pentaphylla*	Circular	290,970
NC_062832.1	*Fragaria tibetica*	Circular	283,001
NC_065239.1	*Fragaria vesca*	Circular	312,993
NC_062592.1	*Fragaria viridis*	Circular	289,289
NC_065219.1	*Fragaria x ananassa*	Circular	285,543
NC_065221.1	*Geum urbanum*	Circular	335,549
NC_065220.1	*Photinia serratifolia*	Circular	473,561
NC_062588.1	*Potentilla micrantha*	Circular	422,985
NC_065228.1	*Prunus armeniaca*	Circular	510,346
NC_065230.1	*Prunus kanzakura*	Circular	422,215
NC_065231.1	*Prunus mira*	Circular	429,732
NC_065232.1	*Prunus mume*	Circular	535,727
NC_065233.1	*Prunus salicina*	Circular	508,005
NC_060491.1	*Prunus salicina x Prunus armeniaca* 'Harmony'	Circular	484,858
NC_065066.1	*Prunus schneideriana*	Circular	434,334
NC_065234.1	*Prunus sibirica*	Circular	510,187
NC_077519.1	*Prunus tenella*	Circular	452,158
NC_065235.1	*Prunus yedoensis*	Circular	456,900
NC_054332.1	*Pyrus betulifolia*	Circular	469,928
NC_065229.1	*Pyrus communis*	Circular	443,525
NC_065227.1	*Pyrus ussuriensis x Pyrus communis* 'Zhongai 1'	Circular	441,853
NC_065218.1	*Pyrus x bretschneideri*	Circular	458,897
NC_065236.1	*Rosa chinensis*	Circular	277,763
NC_077627.1	*Rosa hybrid*	Circular	270,143
NC_065237.1	*Rosa rugosa*	Circular	302,947
NC_065238.1	*Rubus chingii*	Circular	472,138
NC_052880.1	*Sorbus aucuparia*	Circular	384,977
NC_052879.1	*Sorbus torminalis*	Circular	386,758

Table S3. Summary of coverage of mitogenome assembly in *M. baccata* 'ZA'.

Sequencing type	Min	1st quartile	Median	Mean	3rd quartile	Max	Standard deviation	2.5% percentile	97.5% percentile
Illumina PE	383	558	587	604.43	619	10,161	266.5	505	697

Table S4. Annotation information of predicted protein-coding genes in the *M. baccata* 'ZA' mitogenome.

PCG name	Minimum position	Maximum position	CDS length	Genome interval	Strand direction
atp1	148,594	150,126	1,533	1	forward
atp4	324,188	324,784	597	1	reverse
atp6	372,444	373,427	984	1	reverse
atp8	356,878	357,357	480	1	forward
atp9	249,526	249,807	282	1	forward
ccmB	257,220	257,840	621	1	forward
ccmC	265,984	266,736	753	1	forward
ccmFC	321,611	323,882	1,311	2	reverse
ccmFN	231,380	233,152	1,773	1	reverse
cob	197,389	198,570	1,182	1	reverse
cox1	312,096	313,583	1,488	1	forward
cox2	328,598	329,380	783	1	reverse
cox3	357,959	358,756	798	1	forward
matR	87,524	89,491	1,968	1	forward
mttB	314,536	315,255	720	1	forward
nad1	61,741	219,656	978	5	mixed
nad2	78,352	158,270	1,467	5	mixed
nad3	171,752	172,108	357	1	forward
nad4	291,081	299,567	1,488	4	forward
nad4L	324,944	325,246	303	1	reverse
nad5	201,956	282,095	2,013	5	mixed
nad6	151,430	152,047	618	1	forward
nad7	142,213	148,503	1,185	5	forward
nad9	31,271	31,843	573	1	reverse
rpl5	199,912	200,469	558	1	reverse
rpl10	35,567	36,055	489	1	forward
rpl16	4,023	4,430	408	1	reverse
rps1	137,037	137,645	609	1	forward
rps3	354,343	356,007	1,665	1	forward
rps4	135,260	136,318	1,059	1	forward
rps12	172,157	172,534	378	1	forward
rps13	60,499	60,849	351	1	forward
rps14	199,695	199,910	216	1	reverse
sdh3	159,053	159,217	165	1	reverse
sdh4	209,156	209,578	423	1	reverse
sdh4	358,684	359,088	405	1	forward

Table S5. Eight intron-contained mitochondrial genes in *M. baccata* 'ZA' mitogenome.

Gene name	Intron number	Genome location (+/- strand)				
		Exon1	Exon2	Exon3	Exon4	Exon5
ccmFC	1	[323122:323882](-)	[321611:322160](-)			
nad1	4	[219272:219656](-)	[61741:61823](+)	[63270:63461](+)	[86799:86857](+)	[90280:90538](+)
nad2	4	[158118:158270](-)	[156529:156920](-)	[78352:78512](+)	[80360:80932](+)	[82297:82484](+)
nad4	3	[291081:291541](+)	[292958:293472](+)	[296688:297110](+)	[299479:299567](+)	
nad5	4	[250015:250244](+)	[251093:252308](+)	[201956:201977](-)	[281701:282095](-)	[280622:280771](-)
nad7	4	[142213:142355](+)	[143264:143332](+)	[144629:145095](+)	[146151:146394](+)	[148242:148503](+)
trnE-UUC	1	[233211:233243](+)	[234066:234124](+)			
trnM-CAU	1	[102850:102881](-)	[102803:102843](-)			

Table S6. Identified SSRs in mitogenomes of *M. baccata* 'ZA' and others.

Species name/NCBI accession	Number of identified SSRs							
	Total	Compound	Mono-	Di-	Tri-	Tetra-	Penta-	Hexa-
M. baccata 'ZA'/PP826182	115	6	39	21	14	40	1	0
M. baccata/ON478159	121	7	41	22	16	41	1	0
M. domestica/NC_018554	114	5	37	21	14	41	1	0
M. domestica 'Yantai fuji 8'/MN964891	116	5	38	22	14	41	1	0
M. domestica 'Gala'/ON478160	116	5	38	22	14	41	1	0
M. domestica 'Honeycrisp'/OR876282	116	5	38	22	14	41	1	0
M. hupehensis var. mengshanensis/KR534606	125	5	40	24	16	44	1	0
M. kansuensis/MW057419	115	5	38	21	14	41	1	0
M. sieversii/ON478161	117	5	38	21	15	42	1	0
M. sylvestris/ON478162	116	5	38	22	14	41	1	0

Table S7. Different type SSRs in mitochondrion genome of *M. baccata* 'ZA'.

SSR ID	SSR type	SSR	SSR size	SSR start	SSR end
1	p4	(ATAC)3	12	3726	3737
2	p1	(T)10	10	4454	4463
3	p2	(AT)6	12	11252	11263
4	p4	(AGGA)3	12	11468	11479
5	p4	(TGCT)3	12	13130	13141
6	p1	(A)10	10	15332	15341
7	p4	(AAGA)3	12	18877	18888
8	p1	(T)10	10	23954	23963
9	p4	(TTTC)3	12	24075	24086
10	p2	(TA)5	10	29537	29546
11	p1	(A)11	11	30363	30373
12	p4	(CTTA)3	12	30668	30679
13	p3	(GTT)5	15	42882	42896
14	p1	(T)10	10	45105	45114
15	p4	(CGCC)3	12	46122	46133
16	p1	(A)10	10	48755	48764
17	p1	(T)10	10	56035	56044
18	p3	(GTT)5	15	57653	57667
19	p4	(AAGA)3	12	60370	60381
20	p1	(T)12	12	60919	60930
21	p4	(CTAG)3	12	64652	64663
22	p4	(GAAA)3	12	68259	68270
23	p1	(A)10	10	71145	71154
24	p2	(CT)5	10	72727	72736
25	p1	(T)11	11	73620	73630
26	p4	(TGAC)3	12	75186	75197
27	p1	(T)14	14	79662	79675
28	p2	(AT)5	10	81574	81583
29	p4	(GTGA)3	12	85981	85992
30	p1	(A)11	11	86188	86198
31	p1	(T)10	10	86818	86827
32	p4	(AAGA)3	12	87459	87470
33	p4	(AATG)3	12	90091	90102
34	p1	(T)10	10	91937	91946
35	p4	(AAGA)3	12	93022	93033
36	p4	(ATGA)3	12	93212	93223
37	p1	(T)14	14	93540	93553
38	p2	(TC)5	10	98771	98780
39	p3	(TCC)4	12	98951	98962
40	p1	(A)10	10	103833	103842
41	p4	(AAGT)3	12	121756	121767
42	p3	(AAG)4	12	122141	122152

43	p1	(A)12	12	127941	127952
44	p1	(C)10	10	128608	128617
45	p2	(TA)5	10	131704	131713
46	p3	(TTG)4	12	133224	133235
47	p3	(CTT)4	12	137403	137414
48	p1	(T)13	13	137668	137680
49	p4	(GACC)3	12	142579	142590
50	c	(GC)5gtgaaccaacccaacaaacaaggaaagaatgcctctcggggcatctgagaatgattcgag ccgtatgaagggaaactctcacgtacag(T)12(G)12	122	145955	146076
51	p3	(TAA)4	12	158819	158830
52	p4	(TTCT)3	12	163757	163768
53	p1	(A)10	10	164572	164581
54	p2	(AT)5	10	166374	166383
55	p4	(TTTG)3	12	173085	173096
56	p4	(TTCT)3	12	181144	181155
57	p4	(AAAG)3	12	186474	186485
58	p4	(TTGA)3	12	189114	189125
59	p2	(TA)8	16	192735	192750
60	p1	(A)11	11	194481	194491
61	p2	(AG)6	12	194840	194851
62	c	(A)10t(TA)5	21	198635	198655
63	p2	(CT)5	10	203216	203225
64	p3	(CTT)4	12	206742	206753
65	p1	(A)13	13	209863	209875
66	c	(CCTT)3atgagtggaaatccaattccgttttgtcctttttgcataaaaatcaagcta(AT)5	73	220440	220512
67	p1	(A)11	11	222936	222946
68	p1	(A)10	10	224883	224892
69	p4	(TATC)3	12	228396	228407
70	p4	(TCTT)3	12	228552	228563
71	p4	(TTAA)3	12	229540	229551
72	c	(C)10(T)10	20	229713	229732
73	p1	(T)10	10	230821	230830
74	p3	(AGC)4	12	233833	233844
75	p1	(T)10	10	234748	234757
76	p4	(TTTA)3	12	243712	243723
77	p2	(AG)5	10	249035	249044
78	p4	(GCCG)3	12	252962	252973
79	p4	(ACTC)3	12	256589	256600
80	p4	(CTTT)3	12	260007	260018
81	p2	(TC)7	14	264180	264193
82	p3	(CCA)4	12	264536	264547
83	p1	(A)10	10	267791	267800
84	p3	(AGT)5	15	273413	273427
85	p5	(AAGGA)3	15	278140	278154

86	p4	(ATTG)3	12	278525	278536
87	p4	(AGAA)3	12	279127	279138
88	p2	(AC)5	10	279823	279832
89	p3	(CAT)4	12	282327	282338
90	p1	(A)12	12	289252	289263
91	p4	(ATAA)3	12	290824	290835
92	p2	(TC)6	12	292463	292474
93	p1	(T)10	10	303559	303568
94	p2	(AT)5	10	314461	314470
95	p4	(CGGG)3	12	318031	318042
96	p1	(T)10	10	318944	318953
97	p3	(AAG)4	12	320415	320426
98	c	(GAA)4gttccagccaa(T)10	33	320569	320601
99	p4	(GAGC)3	12	328528	328539
100	p2	(TC)6	12	329980	329991
101	p4	(GAAA)3	12	334661	334672
102	p2	(TA)5	10	336015	336024
103	p1	(C)10	10	337968	337977
104	p4	(TTAC)3	12	340400	340411
105	p2	(TA)5	10	345855	345864
106	p1	(A)15	15	346019	346033
107	p1	(G)11	11	351391	351401
108	p4	(CATT)3	12	354548	354559
109	p4	(ATAA)3	12	372496	372507

Table S8. LTRs distribution in *M. baccata* 'ZA' mitogenome.

Genome indices	Period size	Copy number	Consensus size	Percent matches	Percent indels	Score	A	C	G	T	Entropy (0-2)
19126-19179	22	2.5	22	85	8	67	27	9	48	14	1.75
19143-19187	21	2.1	22	87	4	65	26	15	37	20	1.92
42984-43022	18	2.4	15	77	22	51	38	12	33	15	1.85
53940-53977	20	1.9	20	89	10	60	31	34	10	23	1.89
105073-105117	17	2.6	17	82	0	63	31	8	20	40	1.83
114588-114630	18	2.4	18	81	18	54	18	20	25	34	1.96
140430-140463	14	2.5	14	90	4	52	23	14	38	23	1.92
190022-190122	15	6.7	15	96	0	175	27	33	4	33	1.79
190019-190180	15	10.9	15	84	6	122	27	31	6	35	1.81
190016-190133	30	3.9	30	88	4	150	26	33	5	33	1.81
190037-190181	45	3.3	45	82	5	163	27	31	6	34	1.82
190111-190180	29	2.4	29	97	2	131	28	28	5	37	1.8
192719-192755	17	2.3	15	90	9	56	48	0	0	51	1
219670-219748	39	2	39	95	0	140	21	29	18	30	1.97
256063-256088	13	2	13	100	0	52	53	0	23	23	1.46
260224-260266	18	2.6	16	82	10	52	20	20	32	25	1.97
317431-317470	8	4.6	8	82	17	53	47	2	0	50	1.14
317436-317478	19	2.3	19	100	0	86	41	4	0	53	1.21
329677-329732	18	3.1	18	80	10	69	17	17	7	57	1.62
329739-329793	18	3.1	18	97	0	101	5	21	14	58	1.57
329664-329807	36	4.2	35	68	22	99	16	17	9	56	1.65
347086-347120	17	2.1	17	100	0	70	45	28	11	14	1.79

Table S9. The prediction of DRs in *M. baccata* 'ZA' mitogenome.

Repeat position (1)	Repeat position (2)	Repeat length	DR classification	Repeat distance	E-value
191003	311834	429	P	-3	7.22E-240
191017	311828	421	P	-3	4.47E-235
191577	311289	400	P	-3	1.69E-222
191640	311278	348	P	-3	2.25E-191
191655	311270	341	P	-3	3.47E-187
141892	171388	305	F	-2	3.86E-168
191212	311776	278	P	-3	1.60E-149
85019	160644	252	P	-2	2.14E-136
357725	372235	237	P	0	8.07E-133
85042	160644	229	P	0	5.29E-128
48401	264563	203	P	-1	1.45E-109
48435	264563	169	P	0	7.03E-92
200553	249611	175	P	-2	2.35E-90
284496	305858	164	F	0	7.20E-89
200566	249611	162	P	-1	5.60E-85
136820	299532	152	F	-1	5.50E-79
9850	102867	158	F	-3	5.13E-78
209047	359372	145	P	0	1.98E-77
209190	358886	155	P	-3	3.10E-76
85512	160276	150	P	-2	1.94E-75
4462	355848	139	P	-1	3.38E-71
136840	299552	132	F	0	1.33E-69
152725	359498	131	F	0	5.31E-69
141759	171253	135	F	-2	1.69E-66
209181	358915	135	P	-3	2.25E-64
210468	373417	135	F	-3	2.25E-64
89859	228153	121	F	0	5.57E-63
191361	311776	129	P	-2	6.31E-63
141769	171263	125	F	-1	8.15E-63
150486	200471	114	P	0	9.12E-59
307529	353851	107	P	0	1.49E-54
191439	311709	118	P	-3	2.57E-54
311074	325208	114	P	-2	5.29E-54
85561	160276	101	P	0	6.12E-51
311065	325223	108	P	-3	2.06E-48
84869	160941	104	P	-3	4.70E-46
203240	234919	99	P	-3	4.15E-43
209251	358881	99	P	-3	4.15E-43
19459	108893	87	P	0	1.64E-42
103149	355924	86	P	0	6.57E-42
191558	311612	96	P	-3	2.42E-41
191544	311627	95	P	-3	9.37E-41

39175	189691	94	F	-3	3.63E-40
151326	299636	80	F	0	2.69E-38
191910	311266	90	P	-3	8.14E-38
311053	325253	90	P	-3	8.14E-38
203256	234919	83	P	-1	1.05E-37
31853	325256	77	F	0	1.72E-36
59088	264811	77	F	0	1.72E-36
190021	190036	86	F	-3	1.82E-35
190034	190049	84	F	-3	2.71E-34
209166	358981	84	P	-3	2.71E-34
85432	160429	77	P	-1	3.98E-34
191493	311690	83	P	-3	1.04E-33
85820	159575	82	P	-3	4.02E-33
9934	102951	74	F	-1	2.45E-32
203349	234824	70	P	0	2.82E-32
151953	190152	69	P	0	1.13E-31
58966	155621	72	P	-1	3.81E-31
313643	329420	78	P	-3	8.85E-31
31853	311074	66	P	0	7.23E-30
151356	325252	66	P	0	7.23E-30
329885	356760	76	P	-3	1.31E-29
151356	311078	65	F	0	2.89E-29
31847	151356	68	P	-1	9.21E-29
84902	160941	71	P	-2	1.58E-28
60367	87456	67	F	-1	3.63E-28
4462	103172	63	F	0	4.62E-28
356732	373502	73	P	-3	7.41E-28
190021	190051	71	F	-3	1.09E-26
259398	282621	71	F	-3	1.09E-26
14718	282593	64	P	-1	2.22E-26
313658	329420	63	P	-1	8.74E-26
85492	160378	68	P	-3	6.11E-25
59063	59165	61	F	-1	1.35E-24
46005	282593	56	F	0	7.58E-24
329895	373481	66	F	-3	8.93E-24
85724	159704	55	P	0	3.03E-23
85267	160576	58	P	-1	8.24E-23
190034	190064	58	F	-1	8.24E-23
234576	328639	61	F	-2	1.22E-22
14718	46005	64	P	-3	1.30E-22
59071	59173	53	F	0	4.85E-22
152012	189976	52	P	0	1.94E-21
190947	329193	55	F	-1	5.00E-21
14731	225011	51	P	0	7.76E-21

192233	203042	51	F	0	7.76E-21
136820	371774	60	P	-3	2.73E-20
31865	299666	50	P	0	3.10E-20
67364	261601	50	P	0	3.10E-20
299666	311078	50	F	0	3.10E-20
299666	325268	50	P	0	3.10E-20
59036	264761	53	F	-1	7.71E-20
210542	329905	56	F	-2	1.05E-19
50415	259400	49	F	0	1.24E-19
60386	87475	48	F	0	4.97E-19
136924	151326	48	F	0	4.97E-19
46005	225011	51	F	-1	1.19E-18
49846	54425	51	P	-1	1.19E-18
225011	282593	51	F	-1	1.19E-18
190021	190066	56	F	-3	5.67E-18
150600	159165	53	P	-2	6.01E-18
193268	274820	53	F	-2	6.01E-18
299537	371774	55	P	-3	2.15E-17
18406	168580	48	P	-1	7.15E-17
63209	156935	48	P	-1	7.15E-17
192023	311192	51	P	-2	8.90E-17
45184	250068	44	P	0	1.27E-16
85855	159575	47	P	-1	2.80E-16
7669	109282	50	P	-2	3.42E-16
150412	313845	46	F	-1	1.10E-15
64600	227842	52	F	-3	1.16E-15
50415	282623	49	F	-2	1.31E-15
6032	344188	42	P	0	2.03E-15
84943	160920	51	P	-3	4.36E-15
97285	141411	51	F	-3	4.36E-15
45514	58357	44	P	-1	1.68E-14
311144	325208	44	P	-1	1.68E-14
84993	160875	47	P	-2	1.93E-14
49857	54425	40	P	0	3.25E-14
104199	176644	40	F	0	3.25E-14
258154	324136	40	P	0	3.25E-14
85367	160514	49	P	-3	6.18E-14
356767	373491	49	P	-3	6.18E-14
190034	190079	43	F	-1	6.56E-14
293179	330353	46	P	-2	7.40E-14
95934	328467	39	F	0	1.30E-13
190963	329209	39	F	0	1.30E-13
149908	248052	48	P	-3	2.32E-13
259427	282650	42	F	-1	2.56E-13

45800	115363	45	P	-2	2.83E-13
329916	356760	45	P	-2	2.83E-13
4563	355848	38	P	0	5.21E-13
246509	344343	38	F	0	5.21E-13
90563	249859	41	F	-1	1.00E-12
85692	160030	44	P	-2	1.08E-12
10035	103052	46	F	-3	3.26E-12
96878	152698	46	P	-3	3.26E-12
86136	150541	43	F	-2	4.13E-12
86136	200487	43	P	-2	4.13E-12
59190	264811	36	F	0	8.33E-12
152710	221202	36	P	0	8.33E-12
282042	283474	36	F	0	8.33E-12
85381	160504	45	P	-3	1.22E-11
30950	71948	42	P	-2	1.58E-11
144231	224744	35	F	0	3.33E-11
147096	282575	35	P	0	3.33E-11
190116	190145	35	F	0	3.33E-11
112737	159436	44	P	-3	4.55E-11
192046	311176	44	P	-3	4.55E-11
210559	356755	44	P	-3	4.55E-11
86275	91253	38	F	-1	5.94E-11
183244	234091	38	P	-1	5.94E-11
6027	14293	41	F	-2	6.00E-11
10300	280290	41	F	-2	6.00E-11
46563	227806	34	P	0	1.33E-10
50414	109282	34	F	0	1.33E-10
50414	298701	34	P	0	1.33E-10
85702	160030	34	P	0	1.33E-10
96878	144234	34	P	0	1.33E-10
109282	298701	34	P	0	1.33E-10
192001	311222	43	P	-3	1.69E-10
210542	356773	43	P	-3	1.69E-10
110942	116759	40	F	-2	2.28E-10
149916	248052	40	P	-2	2.28E-10
219669	219708	40	F	-2	2.28E-10
299552	371774	40	P	-2	2.28E-10
6033	7682	37	P	-1	2.31E-10
7682	344192	37	F	-1	2.31E-10
193284	274836	37	F	-1	2.31E-10
329738	329756	37	F	-1	2.31E-10
14749	46005	33	P	0	5.33E-10
14749	282593	33	P	0	5.33E-10
109283	259400	33	F	0	5.33E-10

152315	284643	33	F	0	5.33E-10
259400	298701	33	P	0	5.33E-10
10511	234645	42	F	-3	6.30E-10
86499	91463	42	F	-3	6.30E-10
14718	259398	36	P	-1	9.00E-10
21921	319936	36	F	-1	9.00E-10
130329	144232	36	P	-1	9.00E-10
48079	215617	32	F	0	2.13E-09
66274	66735	32	F	0	2.13E-09
85779	159670	32	P	0	2.13E-09
96880	224747	32	P	0	2.13E-09
125519	152710	32	F	0	2.13E-09
125519	221206	32	P	0	2.13E-09
292945	364565	32	P	0	2.13E-09
9773	102794	41	F	-3	2.34E-09
110929	116746	41	F	-3	2.34E-09
190021	190081	41	F	-3	2.34E-09
234614	328677	41	F	-3	2.34E-09
329672	329774	41	F	-3	2.34E-09
28543	372780	38	F	-2	3.29E-09
14299	344194	35	P	-1	3.50E-09
21157	62868	35	P	-1	3.50E-09
96877	130328	35	F	-1	3.50E-09
100973	111032	35	P	-1	3.50E-09
65090	365054	31	F	0	8.53E-09
130334	224745	31	P	0	8.53E-09
199192	221039	40	F	-3	8.68E-09
209320	358871	40	P	-3	8.68E-09
230271	251343	40	P	-3	8.68E-09
31979	329571	37	P	-2	1.25E-08
50414	289365	37	P	-2	1.25E-08
299527	371802	37	P	-2	1.25E-08
14718	50415	34	P	-1	1.36E-08
80506	319293	34	P	-1	1.36E-08
140292	225041	34	F	-1	1.36E-08
147079	152710	34	F	-1	1.36E-08
147079	221204	34	P	-1	1.36E-08
189945	366042	34	P	-1	1.36E-08
144230	221203	39	P	-3	3.21E-08
181827	306843	39	P	-3	3.21E-08
60979	357522	30	P	0	3.41E-08
130334	228097	30	F	0	3.41E-08
144233	228097	30	P	0	3.41E-08
155482	357495	30	P	0	3.41E-08

224746	228097	30	P	0	3.41E-08
46033	259398	36	F	-2	4.72E-08
249749	279489	36	P	-2	4.72E-08
259400	289365	36	P	-2	4.72E-08
14719	109283	33	P	-1	5.28E-08
14719	147049	33	F	-1	5.28E-08
14719	298701	33	F	-1	5.28E-08
137253	137310	33	F	-1	5.28E-08
200839	250245	33	P	-1	5.28E-08
10068	103085	38	F	-3	1.19E-07
62256	288820	38	P	-3	1.19E-07
152309	298636	38	P	-3	1.19E-07
289364	298697	38	F	-3	1.19E-07
5490	14746	35	F	-2	1.78E-07
5490	225012	35	P	-2	1.78E-07
7684	14299	35	P	-2	1.78E-07
109281	289368	35	P	-2	1.78E-07
109282	147048	35	P	-2	1.78E-07
159295	287990	35	P	-2	1.78E-07
6036	287404	32	F	-1	2.05E-07
14752	341087	32	P	-1	2.05E-07
125519	147079	32	F	-1	2.05E-07
150486	249754	32	F	-1	2.05E-07
287404	344194	32	P	-1	2.05E-07
6033	109282	37	F	-3	4.37E-07
109282	344192	37	P	-3	4.37E-07
7685	50414	34	P	-2	6.73E-07
7685	298701	34	F	-2	6.73E-07
14890	75699	34	F	-2	6.73E-07
46035	50415	34	F	-2	6.73E-07
50414	147049	34	P	-2	6.73E-07
55121	298702	34	P	-2	6.73E-07
147049	298701	34	F	-2	6.73E-07
30944	65739	31	P	-1	7.93E-07
76280	89930	31	P	-1	7.93E-07
76280	228224	31	P	-1	7.93E-07
136998	151395	31	F	-1	7.93E-07
151994	190120	31	P	-1	7.93E-07
358911	372205	31	P	-1	7.93E-07
7684	287400	36	P	-3	1.61E-06
46091	59217	36	P	-3	1.61E-06
128964	129655	36	F	-3	1.61E-06
200554	279489	36	F	-3	1.61E-06
7685	259400	33	P	-2	2.53E-06

50414	55122	33	F	-2	2.53E-06
55122	109282	33	F	-2	2.53E-06
84961	160920	33	P	-2	2.53E-06
86508	91472	33	F	-2	2.53E-06
109283	282623	33	F	-2	2.53E-06
147049	259400	33	P	-2	2.53E-06
147049	282623	33	P	-2	2.53E-06
282623	298701	33	P	-2	2.53E-06
284642	298636	33	P	-2	2.53E-06
5495	46006	30	P	-1	3.07E-06
5495	282594	30	P	-1	3.07E-06
15044	46171	30	F	-1	3.07E-06
16030	184340	30	F	-1	3.07E-06
46005	341089	30	F	-1	3.07E-06
46035	109283	30	F	-1	3.07E-06
46035	298704	30	P	-1	3.07E-06
72380	147083	30	P	-1	3.07E-06
85296	160575	30	P	-1	3.07E-06
96882	228096	30	F	-1	3.07E-06
113126	254203	30	F	-1	3.07E-06
135272	136494	30	P	-1	3.07E-06
203206	249023	30	P	-1	3.07E-06
225011	341089	30	F	-1	3.07E-06
282593	341089	30	F	-1	3.07E-06
6032	50413	35	F	-3	5.89E-06
10057	103074	35	F	-3	5.89E-06
50413	344195	35	P	-3	5.89E-06
120331	239706	35	F	-3	5.89E-06
144234	152710	35	F	-3	5.89E-06
221206	224744	35	P	-3	5.89E-06
225041	284642	35	F	-3	5.89E-06
14302	287404	32	F	-2	9.52E-06
14726	259394	32	P	-2	9.52E-06
30953	77395	32	F	-2	9.52E-06
55123	259400	32	F	-2	9.52E-06
3736	283805	34	F	-3	2.15E-05
6033	298701	34	P	-3	2.15E-05
14718	289367	34	F	-3	2.15E-05
96878	221204	34	F	-3	2.15E-05
171690	265841	34	F	-3	2.15E-05
298701	344195	34	F	-3	2.15E-05
4685	284641	31	P	-2	3.57E-05
5454	282630	31	P	-2	3.57E-05
5457	14717	31	F	-2	3.57E-05

31931	311032	31	P	-2	3.57E-05
71648	295125	31	F	-2	3.57E-05
77394	281450	31	F	-2	3.57E-05
97305	141431	31	F	-2	3.57E-05
116216	152713	31	P	-2	3.57E-05
116216	221204	31	F	-2	3.57E-05
190031	190091	31	F	-2	3.57E-05
234624	328687	31	F	-2	3.57E-05
6034	259400	33	F	-3	7.86E-05
7685	14719	33	F	-3	7.86E-05
7686	55122	33	P	-3	7.86E-05
28489	234645	33	P	-3	7.86E-05
47229	63726	33	P	-3	7.86E-05
73043	73053	33	R	-3	7.86E-05
152315	225042	33	F	-3	7.86E-05
259400	344195	33	P	-3	7.86E-05
4599	35594	30	P	-2	1.34E-04
5496	341089	30	P	-2	1.34E-04
46349	63659	30	F	-2	1.34E-04
64526	78064	30	P	-2	1.34E-04
72380	152714	30	P	-2	1.34E-04
72380	221204	30	F	-2	1.34E-04
91174	172140	30	F	-2	1.34E-04
93726	93726	30	P	-2	1.34E-04
112724	249398	30	F	-2	1.34E-04
112732	192263	30	P	-2	1.34E-04
119772	121767	30	P	-2	1.34E-04
142965	201695	30	P	-2	1.34E-04
198180	248100	30	P	-2	1.34E-04
203311	234917	30	P	-2	1.34E-04
209289	372205	30	F	-2	1.34E-04
263605	263605	30	P	-2	1.34E-04
303967	324951	30	F	-2	1.34E-04
14720	55123	32	P	-3	2.86E-04
67438	181564	32	F	-3	2.86E-04
96880	125519	32	P	-3	2.86E-04
125519	144234	32	F	-3	2.86E-04
125519	224747	32	F	-3	2.86E-04
152710	224747	32	F	-3	2.86E-04
187232	206963	32	F	-3	2.86E-04
282627	289365	32	P	-3	2.86E-04
5454	50422	31	P	-3	1.04E-03
5454	259407	31	P	-3	1.04E-03
6035	104197	31	F	-3	1.04E-03

28583	372820	31	F	-3	1.04E-03
29700	265841	31	P	-3	1.04E-03
31845	136998	31	P	-3	1.04E-03
31870	136967	31	P	-3	1.04E-03
39280	189784	31	F	-3	1.04E-03
104197	344196	31	P	-3	1.04E-03
116208	305995	31	F	-3	1.04E-03
116216	147082	31	P	-3	1.04E-03
121626	345645	31	P	-3	1.04E-03
130334	221209	31	F	-3	1.04E-03
136967	151370	31	F	-3	1.04E-03
136967	299680	31	F	-3	1.04E-03
136967	311092	31	F	-3	1.04E-03
136967	325273	31	P	-3	1.04E-03
141397	192459	31	P	-3	1.04E-03
143764	250680	31	F	-3	1.04E-03
150486	279489	31	P	-3	1.04E-03
152028	366008	31	F	-3	1.04E-03
152384	208986	31	F	-3	1.04E-03
181846	370921	31	P	-3	1.04E-03
189981	366008	31	P	-3	1.04E-03
191989	311246	31	P	-3	1.04E-03
192723	192724	31	C	-3	1.04E-03
219286	248139	31	P	-3	1.04E-03
227759	281408	31	F	-3	1.04E-03
281488	328477	31	F	-3	1.04E-03
311140	314408	31	F	-3	1.04E-03
329660	329726	31	F	-3	1.04E-03
313	159074	30	P	-3	3.74E-03
4685	152314	30	P	-3	3.74E-03
4693	344206	30	F	-3	3.74E-03
6041	109290	30	F	-3	3.74E-03
7688	46035	30	P	-3	3.74E-03
7692	14296	30	P	-3	3.74E-03
8507	75194	30	F	-3	3.74E-03
11624	142252	30	F	-3	3.74E-03
14726	50411	30	P	-3	3.74E-03
15096	158608	30	P	-3	3.74E-03
30953	281451	30	F	-3	3.74E-03
45734	54376	30	P	-3	3.74E-03
45747	54363	30	P	-3	3.74E-03
46035	55123	30	F	-3	3.74E-03
46035	147052	30	P	-3	3.74E-03
46039	289367	30	P	-3	3.74E-03

59383	183860	30	F	-3	3.74E-03
59460	233256	30	P	-3	3.74E-03
59488	233176	30	P	-3	3.74E-03
65737	71963	30	F	-3	3.74E-03
72379	125524	30	P	-3	3.74E-03
76214	227893	30	F	-3	3.74E-03
77389	222607	30	P	-3	3.74E-03
90126	234164	30	P	-3	3.74E-03
95936	281480	30	F	-3	3.74E-03
104393	176414	30	F	-3	3.74E-03
109290	344191	30	P	-3	3.74E-03
116218	125521	30	P	-3	3.74E-03
136995	325252	30	P	-3	3.74E-03
144239	359488	30	F	-3	3.74E-03
199205	221052	30	F	-3	3.74E-03
217108	298301	30	P	-3	3.74E-03
221209	228097	30	F	-3	3.74E-03
260503	359772	30	P	-3	3.74E-03
281480	328469	30	F	-3	3.74E-03
311051	329491	30	P	-3	3.74E-03
317435	317454	30	F	-3	3.74E-03

Table S10. Codon usage patterns of mitochondrial protein coding sequences in five *Malus* species.

Species/mtDNA_ID	CAI	CBI	ENC	FOP	A3s	G3s	C3s	T3s	GC3s	GC content
M. domestica/NC_018554	0.168	-0.069	55.59	0.365	0.3577	0.2169	0.2298	0.406	0.357	0.432
M. baccata/NC_065224	0.168	-0.068	55.47	0.366	0.3623	0.2143	0.2298	0.4058	0.355	0.432
M. sieversii/NC_065225	0.167	-0.069	55.54	0.365	0.3602	0.2151	0.2301	0.4059	0.356	0.431
M. sylvestris/NC_065226	0.167	-0.07	55.5	0.365	0.3617	0.2158	0.229	0.4053	0.356	0.431
M. baccata 'ZA'/PP826182	0.166	-0.069	55.59	0.365	0.3623	0.2141	0.2309	0.4036	0.356	0.43

Note: A3s, G3s, C3s, T3s, and GC3s: the base content (A, G, C, T and GC, respectively) of the third codon position (synonymous).

Table S11. Annotated protein coding genes in *M. baccata* 'ZA' cp genome.

Group of genes	Name of genes
Subunits of ATP synthase	*atpA, atpB, atpE, atpF, atpH, atpI*
Subunits of NADH-dehydrogenase	*ndhA, ndhB, ndhC, ndhD, ndhE, ndhF, ndhG, ndhH, ndhI, ndhJ, ndhK*
Subunits of cytochrome b/f complex	*petA, petB, petD, petG, petL, petN*
Subunits of photosystem I	*psaA, psaB, psaC, psaI, psaJ*
Subunits of photosystem II	*psbA, psbB, psbC, psbD, psbE, psbF, psbH, psbI, psbJ, psbK, psbL, psbM, psbN, psbT, psbZ, ycf3*
Large subunit of ribosome	*rpl14, rpl16, rpl2, rpl20, rpl22, rpl23, rpl32, rpl33, rpl36*
Small subunit of ribosome	*rps11, rps12, rps14, rps15, rps16, rps18, rps19, rps2, rps3, rps4, rps7, rps8*
DNA dependent RNA polymerase	*rpoA, rpoB, rpoC1, rpoC2*
Subunit of rubisco	*rbcL*
c-type cytochrom synthesis gene	*ccsA*
Envelop membrane protein	*cemA*
Maturase	*matK*
Protease	*clpP*
Subunit of Acetyl-CoA-carboxylase	*accD*
Conserved open reading frames	*ycf1, ycf2, ycf4*

Table S12. Dispersed repeats classification (P/D) and position in *M. baccata* 'ZA' cp genome.

DR classification	Repeat length	Repeat position (1)	Repeat position (2)	Repeat distance	E-value
P	26354	88318	133848	0	0.00E+00
D	80	39258	39285	-3	1.10E-32
P	63	56091	56091	-1	1.60E-26
P	44	78259	78259	0	2.33E-17
D	53	39258	39312	-3	5.63E-17
P	51	10245	10248	-3	8.00E-16
D	40	102999	125663	0	5.97E-15
P	40	125663	145481	0	5.97E-15
D	39	46845	103001	-1	2.79E-12
P	39	46845	145480	-1	2.79E-12
D	38	46845	125665	-1	1.09E-11
D	32	86806	86838	0	3.91E-10
D	38	62371	62400	-2	6.04E-10
D	34	54722	54761	-1	2.49E-09
D	39	10364	10389	-3	5.89E-09
P	30	8346	48307	0	6.26E-09
D	34	95678	95696	-2	1.23E-07
P	34	95678	152790	-2	1.23E-07
P	34	95696	152808	-2	1.23E-07
D	34	152790	152808	-2	1.23E-07
P	31	201	201	-1	1.46E-07
D	36	39471	39496	-3	2.95E-07
D	33	111950	111981	-2	4.65E-07
P	33	111950	136506	-2	4.65E-07
P	33	111981	136537	-2	4.65E-07
D	33	136506	136537	-2	4.65E-07
D	30	10397	10417	-1	5.63E-07
D	30	33780	33794	-1	5.63E-07
P	33	13980	13980	-3	1.44E-05
D	30	10474	10491	-2	2.45E-05
D	30	84701	84721	-2	2.45E-05
P	30	114207	114207	-2	2.45E-05
D	30	114207	134283	-2	2.45E-05
P	30	134283	134283	-2	2.45E-05
P	32	34491	34501	-3	5.24E-05
D	32	41859	44083	-3	5.24E-05
D	32	93256	93277	-3	5.24E-05
P	32	93256	155211	-3	5.24E-05
P	32	93277	155232	-3	5.24E-05
D	32	155211	155232	-3	5.24E-05
D	31	8345	38422	-3	1.90E-04

D	31	10523	10563	-3	1.90E-04
P	31	10842	10845	-3	1.90E-04
D	31	34478	34501	-3	1.90E-04
P	31	50609	50609	-3	1.90E-04
D	31	62417	62442	-3	1.90E-04
D	30	9880	9901	-3	6.86E-04
D	30	10571	10594	-3	6.86E-04
D	30	10808	10831	-3	6.86E-04
D	30	28921	28945	-3	6.86E-04

Table S13. Identified long tandem repeats in *M. baccata* 'ZA' cp genome.

Cp genome location1	Cp genome location2	Period size	Copy number	Consensus size	Percent matches	Percent indels	Score	Entropy
5049	5077	15	1.9	15	100	0	58	1.27
7102	7127	12	2.2	12	100	0	52	1.47
7144	7177	16	2.1	16	100	0	68	1.24
8674	8704	15	2.1	15	93	0	53	1.7
9888	9931	21	2.1	21	100	0	88	1.05
10269	10396	59	2.2	59	83	10	156	1.02
10268	10481	7	28.6	8	66	21	67	1.12
10398	10447	20	2.5	20	96	0	91	1.16
10316	10432	52	2.2	51	81	18	127	0.98
10328	10481	47	3.4	44	79	17	133	1.12
10372	10474	25	4.1	27	74	20	75	1.17
10328	10481	46	3.4	45	80	12	133	1.12
10321	10481	13	13	13	75	16	103	1.11
10431	10482	27	1.9	27	92	0	86	1.26
10475	10521	17	2.8	17	93	0	76	1.33
10484	10520	10	4	10	80	20	53	1.38
10575	10620	23	2	23	100	0	92	1.39
10618	10651	17	2	17	100	0	68	1.22
10649	10683	11	3.1	11	91	4	52	1.16
10812	10863	23	2.3	23	89	0	86	1.19
13983	14016	17	2	17	100	0	68	1.85
21957	21986	15	2	15	93	0	51	1.88
21962	21991	15	2	15	93	0	51	1.93
28504	28530	13	2.1	13	100	0	54	1.54
28927	28975	24	2	24	100	0	98	1.66
33782	33824	14	3.1	14	100	0	86	1.79
34434	34472	19	2.2	18	90	9	62	0.96
34446	34528	26	3.3	26	82	12	102	0.99
34483	34580	7	12.5	8	67	20	55	1
34486	34532	23	2	23	100	0	94	1
34545	34583	12	3.4	12	82	13	55	1
34474	34583	16	6.8	16	70	20	77	1
34508	34583	38	2	38	85	10	102	1
35163	35214	26	2	26	100	0	104	1.37
35234	35276	18	2.7	15	78	21	52	1.28
35243	35279	18	2.1	18	100	0	74	1.33
38572	38610	19	2.1	19	100	0	78	1.89
39217	39243	12	2.2	12	100	0	54	1.22
39270	39365	27	3.6	27	100	0	192	1.44
39363	39390	11	2.5	11	100	0	56	1.2
39393	39417	12	2.1	12	100	0	50	1.26

39443	39511	22	3	22	87	8	93	0.98
39481	39537	16	3.5	16	80	2	60	0.99
39449	39523	25	3.2	24	90	5	109	0.99
39479	39538	25	2.4	25	91	2	93	0.99
39531	39572	19	2.3	19	95	4	77	1.2
39692	39737	23	2	23	100	0	92	1.77
49397	49438	22	2	20	86	9	57	0.98
49881	49915	18	2	18	88	5	54	1.72
50133	50164	14	2.3	14	100	0	64	1.39
50594	50637	19	2.4	19	85	7	56	1.26
53226	53257	16	2	16	100	0	64	1.67
54898	54926	15	1.9	15	100	0	58	1.37
54919	54986	2	33	2	77	11	66	1.3
54919	54986	14	4.6	14	79	18	75	1.3
54990	55026	13	3	13	84	7	51	1.04
58450	58479	14	2.1	14	93	0	51	1.65
60297	60321	12	2.1	12	100	0	50	1.26
62366	62460	29	3.7	24	78	19	86	1.45
62382	62436	17	3.5	17	76	23	75	1.3
62379	62463	29	3.1	29	90	8	133	1.36
62389	62468	25	3	25	90	7	115	1.43
62913	62955	10	4	10	77	16	50	1.71
62917	62962	23	2	23	100	0	92	1.64
62998	63037	19	2.1	19	100	0	80	1.23
67315	67345	16	1.9	16	100	0	62	1.27
69402	69437	18	2.1	17	94	5	63	1.57
69819	69860	20	2.1	20	100	0	84	1.36
71135	71171	19	2	18	94	5	65	0.67
71830	71860	14	2.2	14	94	0	53	1.16
71949	71987	21	1.9	21	89	5	62	1.86
78437	78472	18	2	18	94	0	63	1.64
83511	83552	21	2	21	81	9	50	1
84702	84745	20	2.2	20	100	0	88	1.33
85792	85828	18	2.1	18	89	0	56	1.04
86807	86870	32	2	32	100	0	128	1.68
89053	89092	20	2	20	95	0	71	1.5
93257	93324	21	3.2	21	82	0	82	1.57
95679	95730	18	2.9	18	94	0	95	1.85
98683	98723	21	2	20	95	4	73	1.22
103170	103208	15	2.9	14	88	11	55	1.12
103212	103237	12	2.2	12	100	0	52	0.77
111951	112014	31	2.1	31	93	0	110	1.87
115341	115371	12	2.7	12	95	5	55	1.38
123441	123479	18	2.2	18	100	0	78	1.62

132504	132537	15	2.3	15	89	0	50	1.09
136507	136570	31	2.1	31	93	0	110	1.87
145284	145309	12	2.2	12	100	0	52	0.77
145313	145351	15	2.9	14	88	11	55	1.12
149798	149838	21	2	20	95	4	73	1.22
152791	152842	18	2.9	18	94	0	95	1.85
155198	155286	21	4.1	21	81	5	81	1.62
159429	159468	20	2	20	95	0	71	1.5

Table S14. Frequency of identified SSR motifs in cp genome of *M. baccata* 'ZA'.

Repeats	5	6	7	8	9	10	11	12	13	14	15	16	17	18	19	20	Total
A	-	-	-	-	-	3	8	3	3	1	-	2	1	3	-	1	25
C	-	-	-	-	-	2	-	-	-	-	-	1	-	-	-	-	3
T	-	-	-	-	-	12	10	2	5	5	1	2	-	2	1	-	40
AT	-	3	-	-	-	-	-	-	-	-	-	-	-	-	-	-	3

Table S15. Population variation statistics of 106 *Malus* species based on mitogenomes.

Data type	Variants	Variation rate	SNPs	INDELs	INS	DEL
High-quality variation data	1,578	1/237	1,424	154	60	94

Table S16. Mitogenome SNP variations in 106 *Malus* species.

Base change/SNPs												Transitions	Transversions	Ts/Tv ratio
AC	AG	AT	CA	CG	CT	GA	GC	GT	TA	TC	TG			
53	80	64	125	30	336	323	38	142	53	93	87	18,852	26,394	0.7143

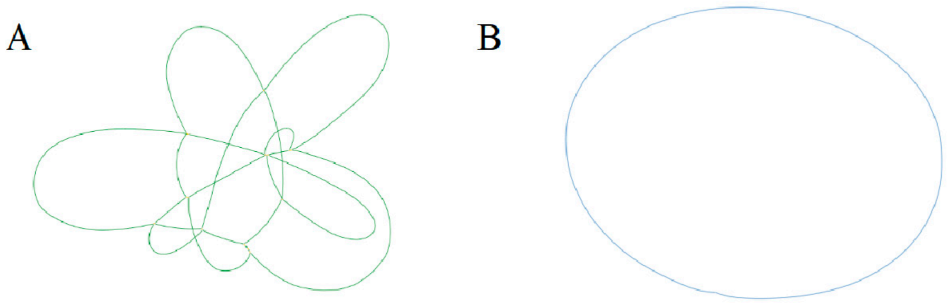

Figure S1. Unicycler assembly process of 'ZA' mitogenome. (A) Assembling of Unicycler and illumina data (overlaps removed graph). **(B)** Mitogenome cyclization (final clean gfa).

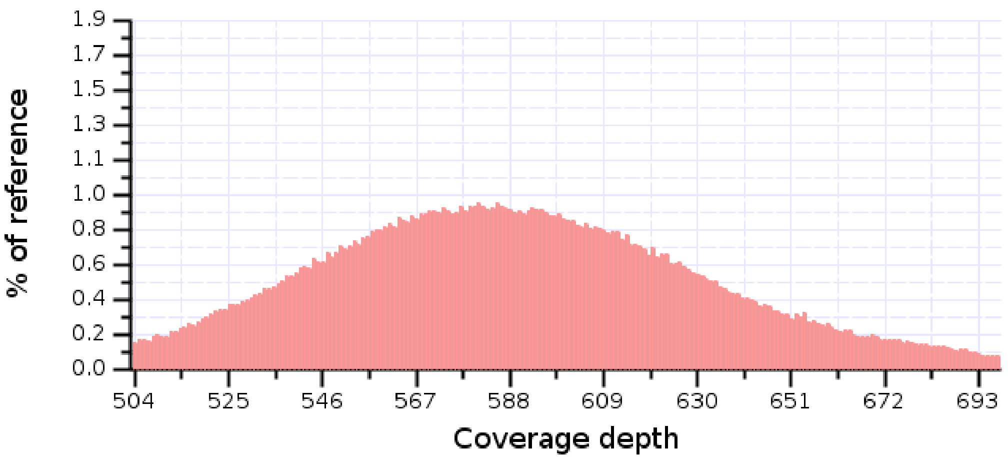

Figure S2. Coverage depth histogram of clean reads for *M. baccata* 'ZA' mitogenome.

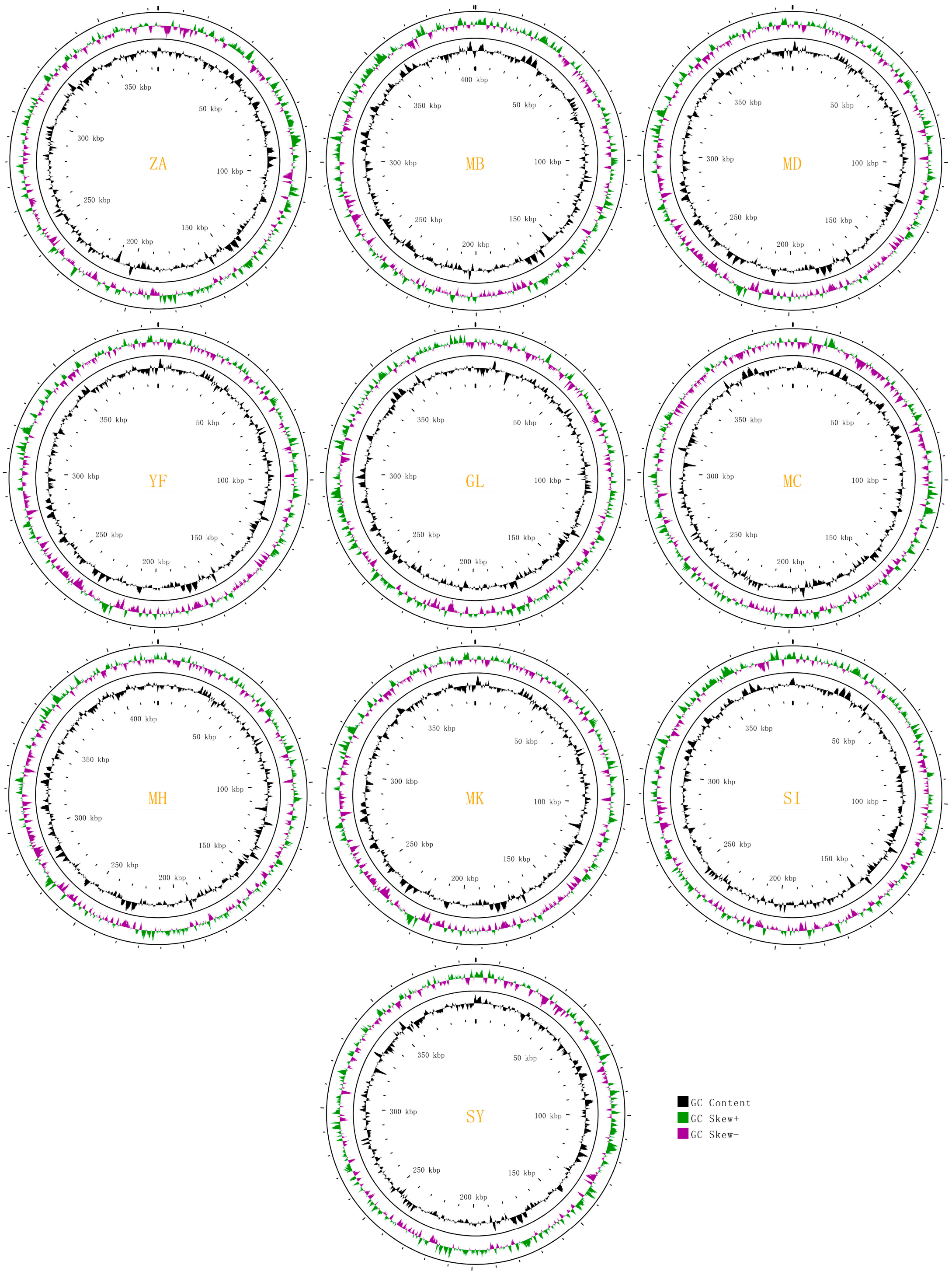

Figure S3. The differences and similarities of GC content/skew between 10 *Malus* mitogenomes. The peaks of GC content inward indicates lower than the average value, and outward indicates higher than the average. GC skew+ means that the content of G is greater than C, and GC skew- is the opposite. The abbreviations of the characters represent the following meanings: ZA: *M. baccata* 'ZA', MB: *M. baccata*, MD: *M. domestica*, YF: *M. domestica* 'Yantai fuji 8', GL: *M. domestica* 'Gala', MC: *M. domestica* 'Honeycrisp', MH: *M. hupehensis* var. mengshanensis, MK: *M. kansuensis*, SI: *M. sieversii*, SY: *M. sylvestris*; and they correspond to those mentioned in Table 1.

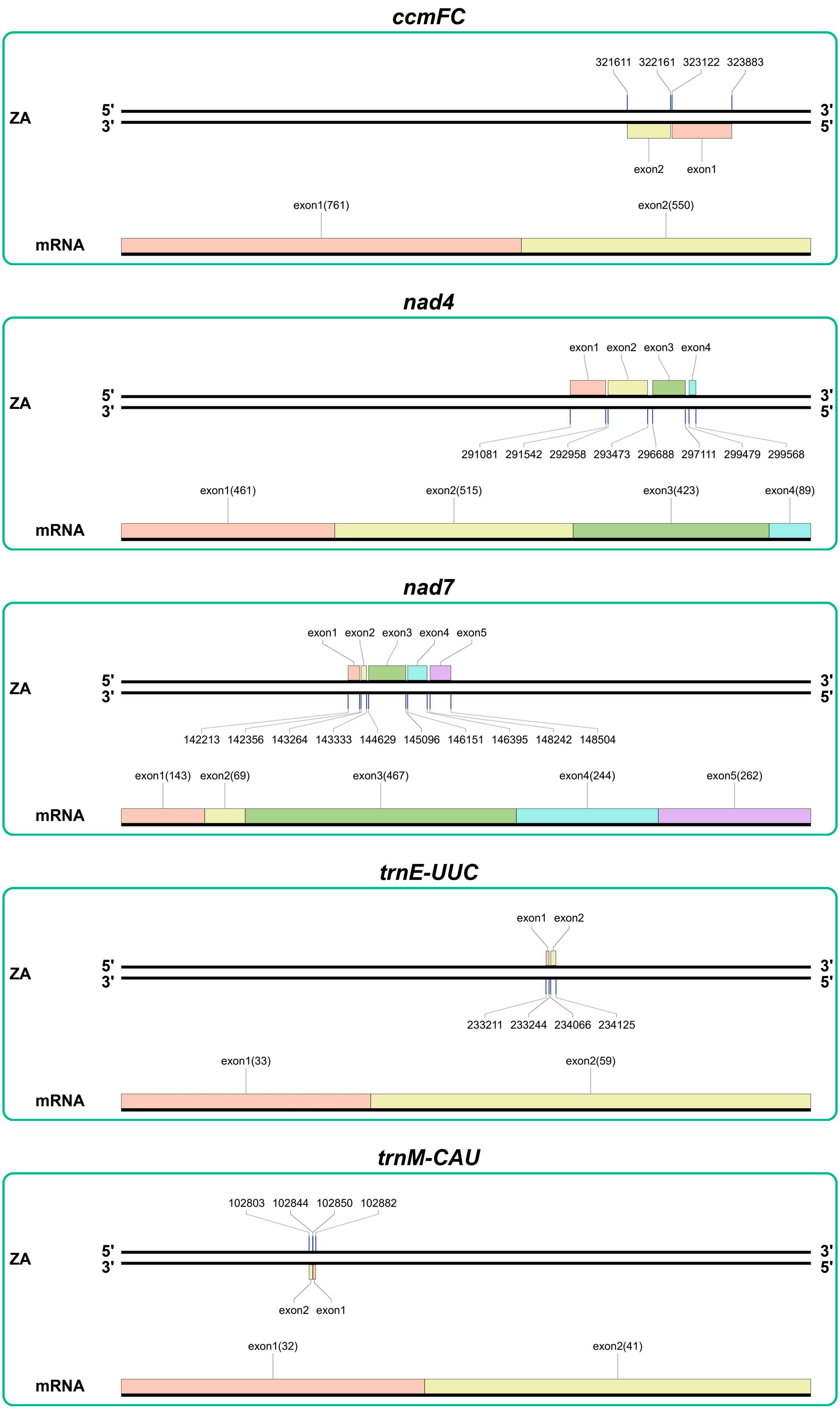

Figure S4. The location and structure of *cis*-splicing genes in *M. baccata* 'ZA' mitogenome.

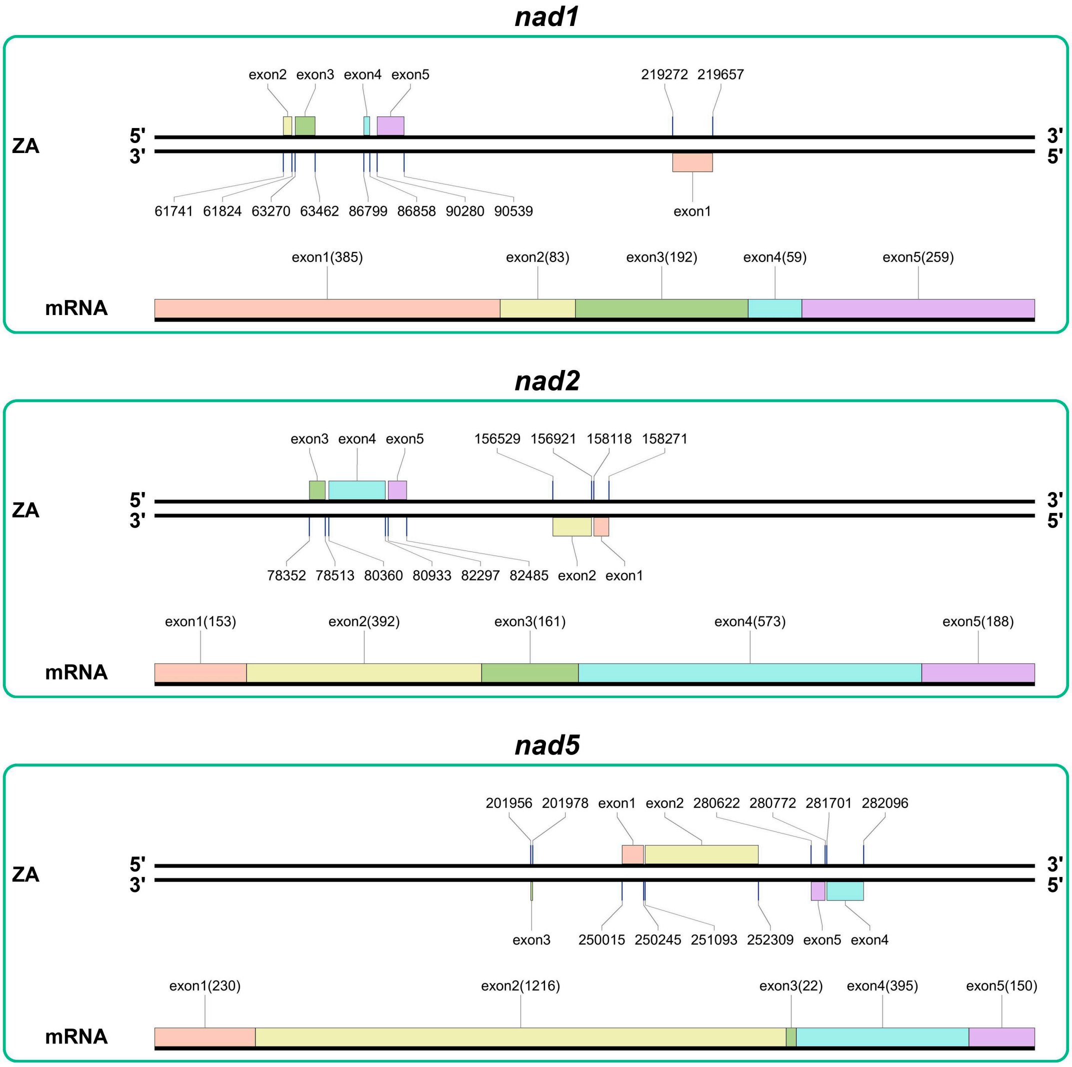

Figure S5. The location and structure of *trans*-splicing genes in *M. baccata* 'ZA' mitogenome.

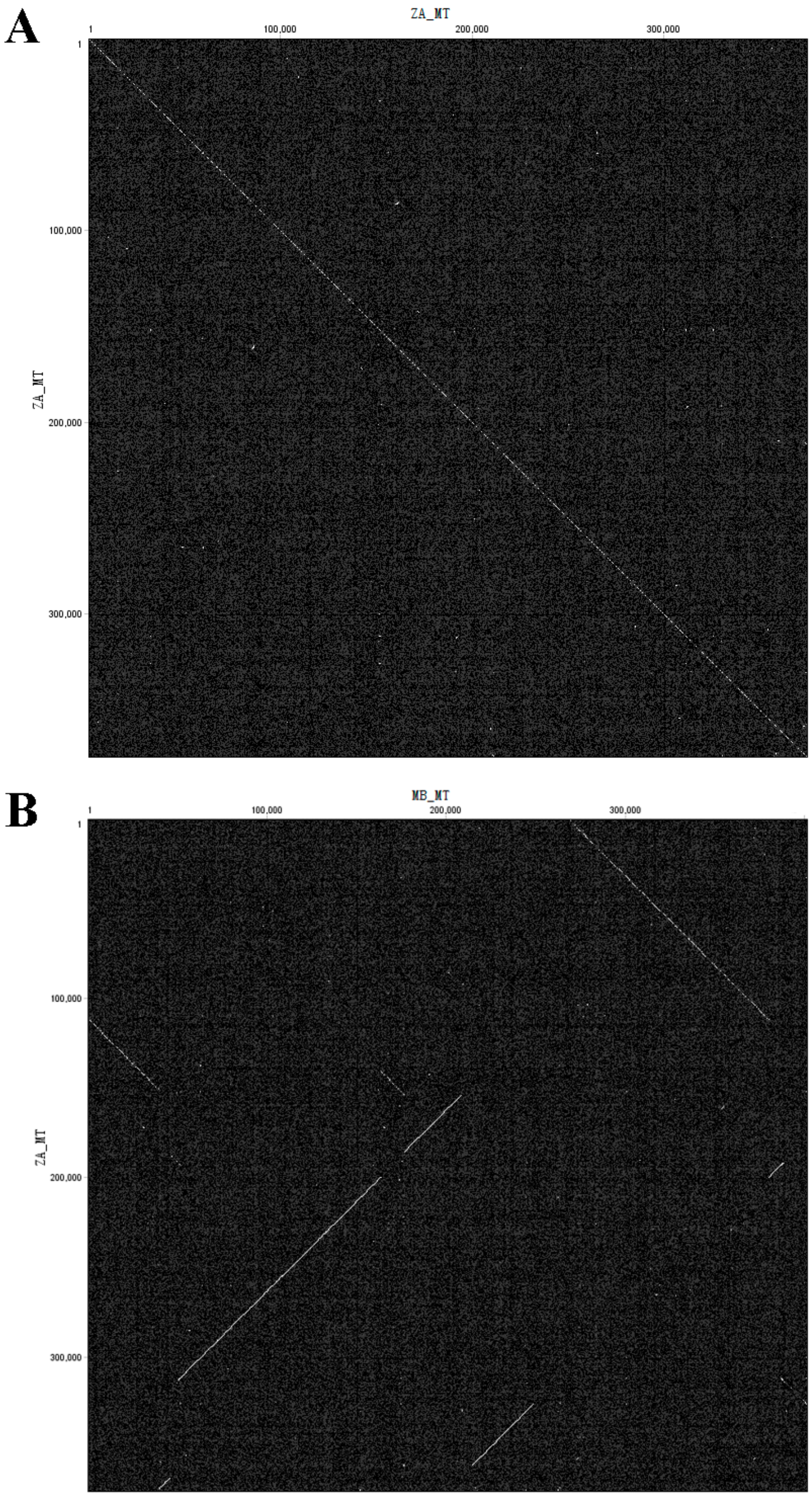

Figure S6. Similarity map based on global alignment of mitogenomes in *M. baccata* and *M. baccata* 'ZA'. (A) Collinearity of 'ZA' based on mitochondrial genome. **(B)** Homology analysis of mitochondrial sequences of *M. baccata* 'ZA' and *M. baccata* (NCBI RefSeq: NC_065224).

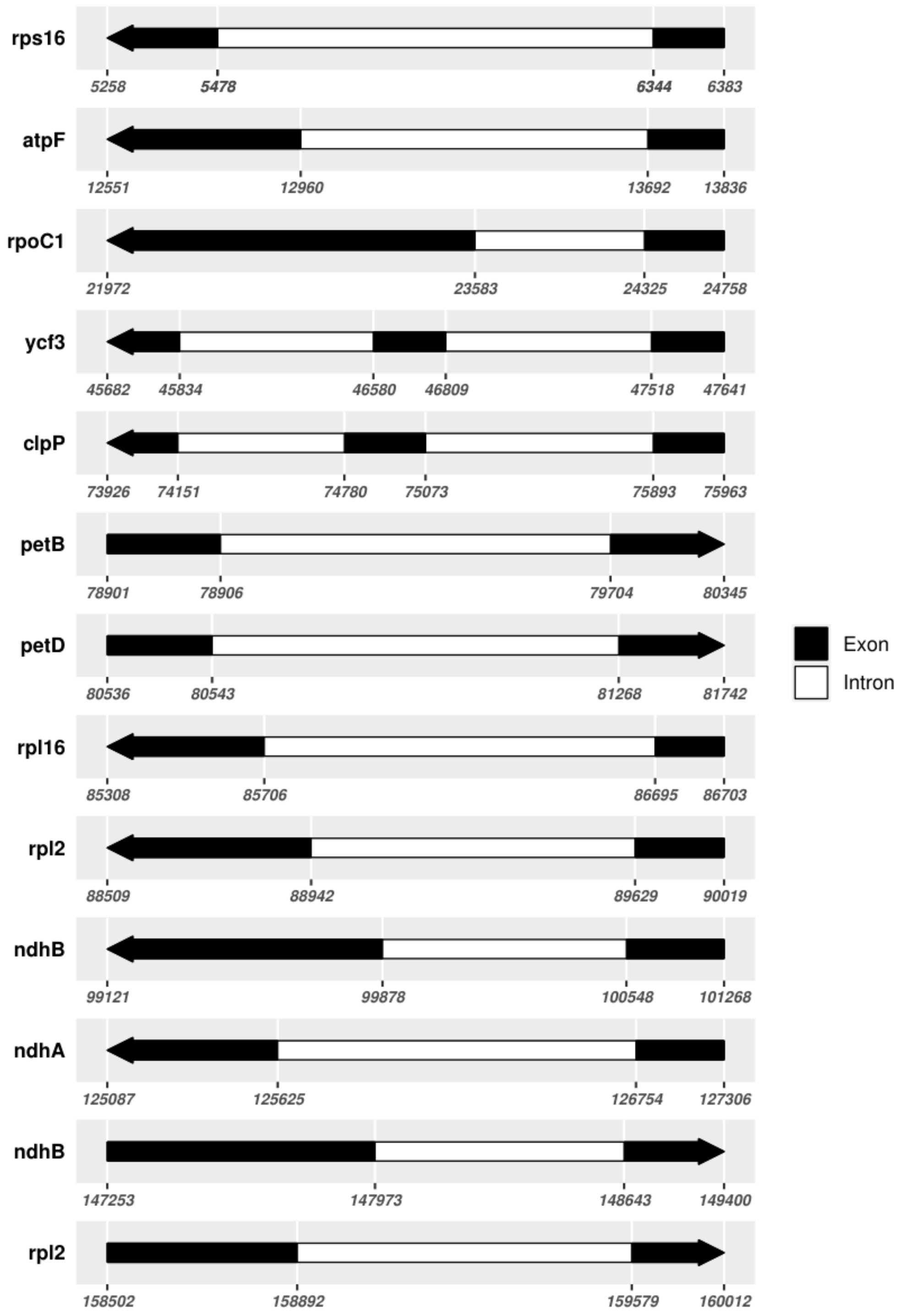

Figure S7. ***Cis*****-splicing genes (exon and intron composition) in the cp genome of *M. baccata* 'ZA'.**

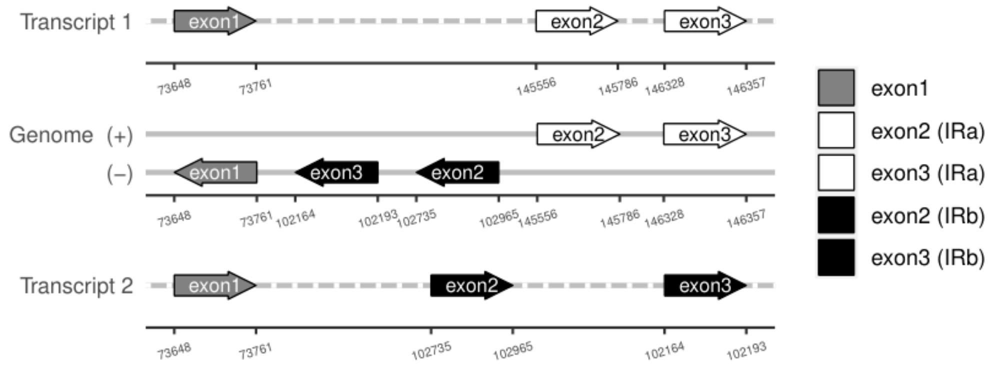

Figure S8. Distribution and position of *rps12* (*trans*-splicing gene) in chloroplast genome of *M. baccata* 'ZA'.
